# A flagella-dependent *Burkholderia* jumbo phage controls rice seedling rot and steers *Burkholderia glumae* toward reduced virulence in rice seedlings

**DOI:** 10.1128/mbio.02814-24

**Published:** 2025-01-27

**Authors:** Brittany S. I. Supina, Jaclyn G. McCutcheon, Sydney R. Peskett, Paul Stothard, Jonathan J. Dennis

**Affiliations:** 1 Department of Biological Sciences, College of Natural & Applied Sciences, University of Alberta, Edmonton, Alberta, Canada; University of Pittsburgh, Pittsburgh, Pennsylvania, USA

**Keywords:** bacteriophage, *Burkholderia*, jumbo phage, rice, flagella, biocontrol, panicle blight

## Abstract

Bacteriophages (phages) are being investigated as potential biocontrol agents for the suppression of bacterial diseases in cultivated crops. Jumbo bacteriophages, which possess genomic DNA larger than 200 kbp, generally have a broader host range than other phages and therefore would be useful as biocontrol agents against a wide range of bacterial strains. Thus, the characterization of novel jumbo phages specific for agricultural pathogens would be of importance for the development of phage biocontrol strategies. Herein, we demonstrate that phage S13 requires *Burkholderia glumae* flagella for its attachment and infection and that loss of *B. glumae* flagella prevents S13 cellular lysis. As flagella is a known virulence factor, loss of flagella results in a surviving population of *B. glumae* with reduced virulence. Further experimentation demonstrates that phage S13 can protect rice plants from *B. glumae*-sponsored destruction in a rice seedling model of infection.

**IMPORTANCE:**

Bacterial plant pathogens threaten many major food crops and inflict large agricultural losses worldwide. *B. glumae* is a bacterial plant pathogen that causes diseases such as rot, wilt, and blight in several food major crops including rice, tomato, hot pepper, and eggplant. *B. glumae* infects rice during all developmental stages, causing diseases such as rice seedling rot and bacterial panicle blight (BPB). The *B. glumae* incidence of rice plant infection is predicted to increase with warming global temperatures, and several different control strategies targeting *B. glumae* are being explored. These include chemical and antibiotic soil amendment, microbiome manipulation, and the use of partially resistant rice cultivars. However, despite rice growth amelioration, the treatment options for *B. glumae* plant infections remain limited to cultural practices. Alternatively, phage biocontrol represents a promising new method for eliminating *B. glumae* from crop soils and improving rice yields.

## INTRODUCTION

*Burkholderia glumae* is a Gram-negative bacterial plant pathogen that infects rice during several developmental stages, causing diseases such as rice seedling rot and bacterial panicle blight (BPB) (1). Yield reductions ranging from 17.7%–75% have been attributed to *B. glumae*, and the incidence of infections is predicted to increase with warming global temperatures (2, 3). For instance, in the United States alone, increases of 1–3°C are projected to cause BPB-related losses equivalent to the amount of rice consumed by 2.17–3.98 million people per year (2). Thus, *B. glumae* represents a major threat to rice production, requiring immediate attention.

Unfortunately, there are limited methods for controlling *B. g*lumae. In the past, a quinolone antibiotic, oxolinic acid, was used to control *B. glumae* in Asia*,* but *B. glumae* resistance to this compound has been reported (1, 4, 5), threatening the efficacy and sustainability of this control strategy. More recently, microbes naturally present in rice seeds and amended soil environments have been explored as potential biocontrol agents (BCAs) for *B. glumae* (6, 7). However, given the potential impacts of BCAs on the soil microbiome, plants, and humans, and the variable efficacy of BCAs against agricultural pathogens (8), this strategy requires more investigation. Therefore, treatment options for *B. glumae* infections remain limited, and new strategies for controlling this pathogen are needed.

Naturally occurring viruses that specifically infect bacteria (bacteriophages, or phages for short) have gained interest for combatting several important crop pathogens including *B. glumae* (9–13). In comparison to chemical treatment options, which may bioaccumulate in the soil, phages are environmentally ubiquitous and amplify in target bacteria, auto-dosing based on host availability (14). Furthermore, during the initial stages of phage infection, phages adhere to surface exposed-structures on their bacterial hosts as receptors (15, 16). This irreversible binding triggers the injection of the phage genome into the host bacterium (15). The exquisite specificity of this phage-receptor interaction limits phage predation to bacterial hosts with compatible receptors, making phages desirable for targeting specific pathogens in complex microbial communities (14) while leaving beneficial microbes unharmed.

Although the absence of a compatible receptor for phage binding represents a major determinant of phage host range, the sensitivity of pathogens to certain phages may be affected by other factors. Specifically, even if successful receptor-mediated host recognition occurs, diverse anti-phage defense systems that detect phage nucleic acids, phage proteins, or other phage signals inside the host cell may stop phage replication (17). Furthermore, in previously susceptible bacterial hosts, spontaneous mutations resulting in lost, modified, or downregulated phage receptors can give rise to bacterial populations lacking compatible receptors for phage adsorption (16). Although detrimental for phage-mediated bacterial killing, these mutations may be exploited therapeutically; phages recognizing virulence factors as receptors may select for resistance by receptor loss, thus steering bacterial populations toward reduced virulence, reduced fitness, or increased antimicrobial susceptibility (16, 18–20).

Phage steering has garnered significant interest in recent years, and the cost of phage-driven receptor loss has been investigated for pathogens including *Burkholderia cenocepacia* (18), *Pseudomonas aeruginosa* (19), and *Pectobacterium carotovorum* (20). Recently, Ruest et al*.* found that in *B. cenocepacia* K56-2, phage-resistance to lipopolysaccharide (LPS) O-antigen or inner core-binding phages resulted in truncated LPS and sensitization to the cationic peptide, colistin (18). Notably, LPS has been identified as a receptor for other phages infecting *Burkholderia cenocepacia, Burkholderia contaminans,* and *Burkholderia psuedomallei* (21–23). In addition to LPS, capsular polysaccharide (CPS) has been identified as a possible receptor for phage ΦBp-AMP1 and other P2-like phages infecting *B. pseudomallei* (23, 24). However, despite these advances in understanding phage-host interactions within *Burkholderia* species, no receptors have been identified for phages infecting *B. glumae*, and the interplay between phage resistance and *B. glumae* virulence remains unstudied.

In this research, we describe the isolation of the novel *Burkholderia-*specific jumbo phage vB_BgluM-SURPRISE13 (S13) and investigate its antimicrobial and anti-virulence activities against *B. glumae*. We identify S13 as the first known *Burkholderia* phage to use flagella as a receptor. Investigation of the *in vitro* and *in planta* antimicrobial activities of S13 show that S13 receptor loss negatively impacts *B. glumae* virulence in rice seedlings. Together, our findings shed light on the applications of flagella-dependent jumbo bacteriophages for agricultural diseases.

## MATERIALS AND METHODS

### Bacterial strains and culture conditions

The bacterial strains used in this study are listed in Table 2 and Table S1. Strains were routinely cultured at 30°C in ½ strength Lennox (½ LB; 10 g/L tryptone, 5 g/L yeast extract, 5 g/L NaCl) broth or media unless otherwise stated. Plates were incubated for 48 h to obtain single colonies, and liquid cultures were grown aerobically with shaking at 225 RPM for 18 h. Medium was supplemented with 25–35 µg/mL chloramphenicol (Cm), 100 µg/mL trimethoprim (Tp), or 100–150 µg/mL tetracycline (Tc) for cloning and plasmid maintenance. Data analysis was conducted using GraphPad Prism 10 for macOS (version 10.1.1).

### Phage isolation, purification, and propagation

Phage isolation was conducted using established soil enrichment protocols (25). Briefly, 10 mL of soil samples was incubated overnight with 1 mL *B. glumae* LMG 2196 culture, 10 mL ½ LB broth, and 1 mL phage suspension media (SM) (50 mM Tris–HCl [pH 7.5], 100 mM NaCl, 10 mM MgSO_4_) at 30°C with shaking at 225 rpm. The enriched soil sample was centrifuged to precipitate soil particles, and the supernatant was passed through a MILLEX-HA 0.45 uM syringe-driven filter (Millipore, Billerica, MA, USA). In addition, 300 μL of the resulting supernatant was combined with 100 μL of LMG 2196 culture and 3 mL of ½ LB soft agar to produce soft agar overlays. S13 was purified from smaller phage particles in the lysate using a cesium-chloride density gradient (22), with modifications. In the absence of a ghost band, 1 mL aliquots were extracted from the top third of the gradient and loaded into 12 kDa molecular weight cutoff dialysis tubing. Samples were dialyzed in suspension media (SM) at 4°C with the SM solution changed every 24 h for 4 days. After dialysis, 100 μL of each aliquot was combined with 100 μL LMG 2196 culture in soft-agar overlays with ½ LB media containing 0.35% (wt/vol) agar. Single plaques were picked from three consecutive overlays producing an axenic stock. Routine S13 propagation and titer determination was conducted using soft-agar overlays (0.35% [wt/vol] agar) of LMG 2196 as described (26, 27).

### Host range, stability, and electron microscopy

The host range of S13 was determined against a panel of 46 clinical and environmental *Burkholderia* isolates, and one *Ralstonia pickettii* strain (see Table 2). Five microliters of serially diluted phage lysate was spotted onto ½ LB (0.3% [wt/vol]) soft agar overlays containing 100 µL overnight culture of each strain in biological and technical triplicates. Plaques or clearing were enumerated after overnight incubation at 30°C. Efficiency of plating (EOP) was calculated as the number of plaques on each strain divided by the number of plaques on LMG 2196, with EOP values above 0.5 considered highly productive phage infection, and EOP values between 0.1 and 0.5 indicating moderate phage productivity (28). EOP values below 0.001 were not considered to be productive phage infection (28). In the absence of plaques, phage activity was quantified by the lowest dilution of S13 producing lysis/clearing of the bacterial lawn. Clearing at 10^4^ PFU/mL or less was arbitrarily considered strong activity, clearing at 10^5^–10^6^ PFU/mL represented moderate phage activity, and clearing or thinning of the bacterial lawn at 10^7^ PFU/mL or higher was considered weak activity.

To assess S13 temperature stability, microcentrifuge tubes filled with 900 µL of SM were incubated for 1 h at 4, 22 (room temperature), 30, 37, or 42°C. Next, 100 µL of the S13 lysate was added to each tube to achieve a final concentration of 1 × 10^6^ PFU/mL. After gentle mixing, a 150 µL aliquot was removed from each tube for initial (T = 0) titer determination. Tubes were statically incubated at the desired temperatures, and the titer was determined after 24 h, 48 h, and 1 week. For each temperature, PFU/mL was calculated from the average titer from three samples, each with three technical replicates.

For transmission electron microscopy (TEM), S13 was propagated using ½ LB top agarose, producing clean, high-titer lysates. Ten microliters of lysate were loaded onto a carbon-coated copper grid, stained with 4% uranyl acetate, and visualized using a Philips/FEI Morgagni transmission electron microscope with charge-coupled device camera at 80 kV (U. Alberta Department of Biological Sciences Advanced Microscopy Facility). Capsid diameters and uncontracted tail lengths were measured using ImageJ (29) software (*n* = 6).

### Growth reduction assays and growth curves

Growth reduction assays were conducted according to published protocols (22). Briefly, *B. glumae* LMG 2196 was sub-cultured 1:100 in ½ LB at 30°C or 37°C with shaking (225 RPM) and standardized to optical density at 600 nm (OD_600_) = 0.1. Standardized sub-cultures were diluted 1:100 in ½ LB to achieve an approximate cell concentration of 1 × 10^6^ CFU/mL. Phage lysate was serially diluted in ½ LB, and 100 µL of the diluted lysate was combined with 100 µL of the diluted inoculum in a 96-well plate, producing MOI (multiplicity of infection) values of 0.1–100. The 96-well plate was incubated at 30°C or 37°C in an Epoch 2 Microplate Spectrophotometer (BioTek) with shaking at 237 cpm, and optical density (OD_600_) was measured every 30 min for 36 h. The concentration of each diluted inoculum was determined using the track method to verify MOI (30) . To compare phage activity across conditions, the area under the curve (AUC) at each MOI was divided by the AUC of the no-phage control for each temperature. This value was subtracted from 1, producing the growth reduction coefficient (GRC) (31). Growth reduction results represent the average of four biological replicates.

Bacterial growth curves were conducted as previously described (18, 22). Overnight cultures of each strain were standardized to an OD_600_ of 0.1 and diluted 1:100 into fresh LB media in a 96-well plate to a final volume of 200 µL. The plate was incubated at 30°C in an Epoch 2 Microplate Spectrophotometer (BioTek) with shaking at 237 cpm. Optical density (OD_600_) was measured once every hour for 36 h. The results show the average measurements from three biological replicates with two technical replicates each.

### Phage genome assembly and annotation

Phage gDNA was isolated using a phenol/chloroform extraction method (32). Library preparation and sequencing was conducted by SeqCenter (Pittsburgh, PA) using an Illumina DNA Prep kit and the Illumina NextSeq 2000 platform. Illumina paired-end reads were assessed for quality with FastQC (v.0.12.1) (33) and trimmed using Trimmomatic (v.0.39) (34) with 92.83% of 1,454,350 input read pairs surviving. Trimmed reads were assembled with Unicycler (v.0.4.8) (35), producing a large contig of 227,647 bp with an average of 1661.8× coverage. Quality analysis using CheckV (v.1.0.1) against the checkv db-v1.5 (36) confirmed a complete, high-quality genome assembly. Regions of lower coverage and the ends of the contig were verified through Sanger sequencing of PCR products obtained using seven distinct primer sets (Table S2). Closely related phage genomes were identified through a Blastn search of taxid:2731619 (Myoviridae), and the average nucleotide identity shared between S13 and similar phages RSF1 (NC_028899.1), RSL2 (NC_028950.1), FLC6 (LC592711.1), and FLC8 (LC667450.1) was calculated using FastANI (v.0.1.3) accessed through the Kbase data platform (37, 38). In the absence of confirmed termini, the genome start site was determined through whole genome alignment with the related *Chiangmaivirus* type strain RSF1 (NC_028899) using the Mauve plugin for Geneious Prime (39).

Structural annotation was completed using the Glimmer3 plugin for Geneious Prime (40), GenemarkS (v.4.28) (41), and Prokka (v.1.14.6) (42), followed by manual curation of putative protein coding sequences (43). Putative protein functions were assigned through NCBI Blastp searches against taxid 10293 (viruses) and taxid 2 (bacteria) with an E value cutoff of 1.0E^−3^. Functional annotations were supported, and additional annotations were assigned using the InterProScan plugin for Geneious Prime (44), employing NCBIFAM, CDD, PfamA, SignalP_EUK, SignalP_Gram_Negative, Panther, and Superfamily applications. Protein sequence similarity searches using phmmer and hmmscan (HmmerWeb v.2.41.2, https://www.ebi.ac.uk/Tools/hmmer/) were used to further investigate some functional annotations. ARAGORN (v.1.2.41) (45) and tRNAscanSE (v.2.0) (46) were used to screen for phage-encoded tRNAs (accessed 23 March 2024).

### Phage-resistant mutant isolation and genome analysis

S13-resistant (S13^R^) mutants were isolated as previously described (18), with some modifications. Overnight cultures of *B. glumae* LMG 2196 or AU6208 were diluted 1:1,000 in ½ LB. Next, 150 µL of high-titer S13 lysate (5 × 10^9^ PFU/mL) was combined with 150 µL of diluted overnight culture and incubated at 30°C with shaking (225 RPM) for 30 min. Tubes containing sterile SM in place of phage lysate were used to obtain *B. glumae* colonies evolved under isolation conditions as controls. After incubation, 1.5 mL of SM and 15 mL of ½ LB were added to each tube, followed by incubation with shaking for 48 h at 30°C. Surviving bacterial cells were pelleted at 6,800 × *g* and washed three times in ½ LB to remove phage. Washed cells were diluted, spread onto ½ LB plates, and incubated at 30°C. After 2 days, single colonies of surviving bacterial cells or controls were isolated and saved for further analysis.

To confirm S13-resistance, soft agar overlays made with each isolate were spotted with 10 μL of 1 × 10^8^ PFU/mL S13 lysate and observed for clearing or thinning of the bacterial lawn after overnight incubation at 30°C. For each *B. glumae* strain, three fully resistant mutants were randomly selected for additional characterization. Bacterial gDNA from S13^R^ mutants and WT AU6208 or LMG 2196 was sequenced by SeqCenter (Pittsburgh, PA), with library preparation completed using an Illumina DNA Prep kit and sequencing completed on an Illumina NextSeq 2000 platform. Genetic variations in S13^R^ mutants were predicted using snippy (Galaxy v.4.6.0) (47) and BreSeq (v.0.38.2) (48), in comparison to the LMG 2196 reference genome (accession GCF_000960995.1), or AU6208 reference contigs assembled from WT sequencing reads with Unicycler (v0.4.8) (35) and annotated with Prokka (v1.11) (42). For LMG 2196 isolates, BreSeq analysis was completed by SeqCenter (Pittsburgh, PA) using Breseq v0.36.1 (Pittsburgh, PA). Both mutation predictions and marginal predictions computed by Breseq were considered in our analysis.

### Strain construction and complementation

Clean deletions of flagella associated genes *fliE, flgK,* and *flgC* were generated through homologous recombination using vectors pGPI-SceI and pDAI-SceI as previously described (49). Regions flanking each gene were PCR-amplified using Up or Down primer sets (Table S2), producing upstream and downstream fragments with 15 bp overlapping sequences at the 5’ ends. Fragments with the expected size of ~1,000 bp were gel extracted using a QIAquick gel extraction kit (Qiagen, Inc., Germantown, MD, USA). To create DNA fragments containing the desired in-frame deletions, the upstream and downstream fragments for each gene were joined in an overlap extension PCR using primers containing recognition sites for the restriction enzyme *KpnI* or *XbaI* (Table S2). These fragments were each gel extracted and cloned into pGPI-*SceI* using restriction cloning methods. Ligated DNA was transformed into chemically competent *Escherichia coli* DH5α cells, and transformants were selected on LB agar with 100 µg/mL trimethoprim (Tp). Deletion vectors were isolated using a QIAquick miniprep kit (Qiagen, Inc., Germantown, MD, USA) and transformed into electrocompetent LMG 2196 or AU6208 using a Bio-Rad MicroPulser (Bio-Rad, Hercules, CA, USA). Tp-resistant colonies resulting from the integration of the deletion vector into the bacterial chromosome were selected on LB + 100 µg/mL Tp and confirmed with PCR. To initiate a second crossover event, cells harboring the integrated deletion vectors were transformed with pDAI-*SceI* and selected on LB supplemented with 150 µg/mL tetracycline (Tc) for LMG 2196 and 100 µg/mL Tc for AU6208. Tc-resistant colonies were replica plated onto LB + 100 µg/mL Tp. Tp-sensitive colonies emerging after 72 h at 30°C were screened for the mutation using *fliE, flgC,* or *flgK*-specific complementation primers (Table S2). Confirmed deletion mutants were passaged 1–3 times on LB at 30°C to cure the pDAI-*SceI* plasmid.

Genetic complementation of *flgC, flgK,* or *fliE* in the constructed mutants was achieved using the broad-host range expression vector, pBBR1-MCS (50). Each wildtype gene was PCR amplified from AU6208 or LMG 2196 using the complementation primers listed in Table S2. The PCR-amplified fragments were purified, digested with the appropriate restriction enzymes, and ligated into pBBR1-MCS. Ligation mixtures were transformed into chemically competent *E. coli* DH5α, and transformants were selected on LB + 35 µg/mL Cm. The complementation vectors were isolated using a QIAquick miniprep Kit (Qiagen, Inc., Germantown, MD, USA) and verified with Sanger sequencing. After verification, complementation vectors or empty pBBR1-MCS were electroporated into AU6208 or LMG 2196 followed by selection on LB + 35 µg/mL Cm.

### Swimming motility and biofilm assays

To indirectly screen for functional flagella, swimming motility assays were conducted as described (51) with minor modifications. Fresh ½ LB agar plates were prepared at a concentration of 0.25% (wt/vol) agar for LMG 2196 or 0.35% (wt/vol) agar for AU6208 and dried for 3 h prior to use. When required, the plates were supplemented with 25–35 µg/mL Cm. Overnight cultures of each strain were grown at the desired temperature and standardized to an OD_600_ of 1.0. Two microliters of standardized inoculum were injected half-way into the fresh agar plates. Plates were incubated at 30°C or 37°C for 24 h, after which the swimming zone diameters were measured using Tresna Instruments digital calipers (Tresna, Guangxi, China). Swimming diameter measurements represent the average of nine swim zones measured from three biological replicates with three technical replicates each.

Biofilm formation was quantified following published protocols (52) with modifications. Overnight cultures grown in LB were sub-cultured 1:50 into fresh LB media and grown at 30°C for 2 h and 45 min. Sub-cultures were diluted 1:50 into LB +2.5% (wt/vol) dextrose, and 2 mL of each culture was incubated statically in a 24-well flat-bottom polystyrene plate (Corning Inc., Kennebunk, ME, USA) at 30°C for 48 h. After 48 h, surface-localized biofilms were imaged using an Apple iPhone 14. After imaging, the culture was removed, and the wells were gently washed with 2 mL of 0.9% (wt/vol) NaCl to remove non-adherent cells. The wells were stained with 2 mL of 0.1% (wt/vol) crystal violet for 15 min, followed by three washes. For each wash, 2 mL of milliQ H_2_O was added to each well, and the plates were incubated on the bench for 5 min before gentle removal of wash solution. The plate was dried at room temperature for 30 min. Once the plate was dry, the contents of each well were solubilized in 1 mL of 30% glacial acetic acid, and 200 μL from each well was transferred to a 96-well plate for absorbance determination at 570 nm in a Wallac 1420 Victor^2^ microplate reader (Perkin Elmer, Waltham, MA, USA). Experiments were conducted in biological triplicate with two technical replicates each.

### Evaluation of phage rescue and bacterial virulence in rice seedlings

Rice seedling rot experiments were conducted similar to other studies (7, 11, 53), with modifications. Rice (*Oryza sativa*) “Zerawchanica” seeds (Annapolis Seeds, Nictaux, NS. Canada) were surface sterilized in 10% bleach for 20 min, washed four times in sterile milliQ H_2_O, and dried overnight at room temperature. Overnight cultures of *B. glumae* AU6208 were standardized to OD_600_ = 0.1 and diluted in sterile milliQ H_2_O to achieve a final average inoculum of ~2 × 10^5^ CFU for phage rescue experiments, and ~6 × 10^4^ CFU for bacterial virulence experiments, which were confirmed through CFU counts. Seeds were incubated in diluted inoculum or milliQ H_2_O at 30°C for 4 h with shaking at 225 rpm. After inoculation, seeds were washed once in sterile milliQ and dried overnight. Inoculated seeds were germinated in the dark at 30°C in 10 mL sterile milliQ H_2_O with or without S13 lysate added to a final concentration of 10^8^ PFU/mL. After 48 h, seeds were sown in 7.2 × 11.5 x 4.2 cm plastic containers containing autoclave-sterilized commercial potting soil (Jiffy Group, Zwijndrecht, Netherlands) pre-wetted with 60 mL of sterile milliQ. Seeds were incubated at 30°C in a humid Sunblaster TH50 Mini Greenhouse (SunBlaster Holdings ULC, Langley, BC, Canada) under 14 h:10 h light:dark conditions. As indicators of *B. glumae* infection (54), seedling height and root lengths were recorded 10 days after sowing for phage rescue experiments, and 9 days after sowing for bacterial virulence experiments. Three replicate experiments were conducted, each with 18–21 seeds per treatment group. Statistical significance was determined using a nested one-way ANOVA at *P* = 0.05. For phage experiments, pairwise comparisons between all treatment groups were conducted using Tukey’s multiple comparison test, whereas virulence of *B. glumae* strains was determined in comparison to WT AU6208 using a Dunnett’s multiple comparison test.

## RESULTS AND DISCUSSION

### Genomic analysis indicates that phage S13 is a novel jumbo bacteriophage

**Fig 1:**
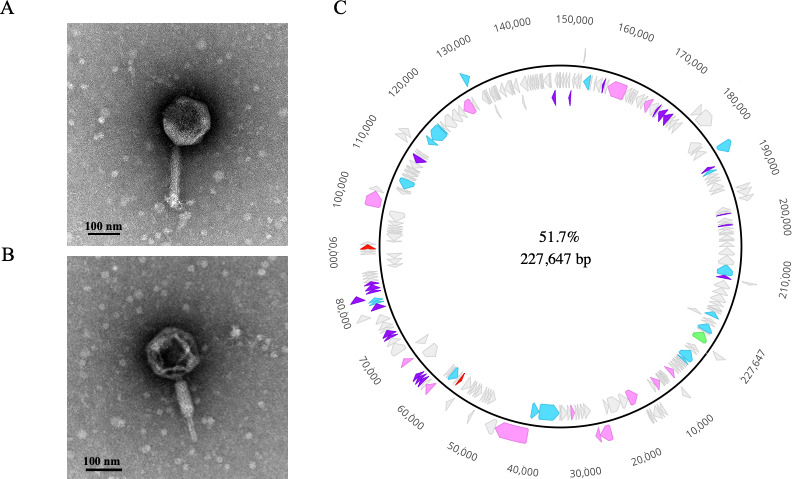
Structural and genomic characterization of S13 phage. (**A and B**) Electron micrographs of S13. Phage particles were stained with 4% uranyl acetate on a copper grid, and viewed at ×110,000 magnification. Micrographs depict non-contracted (**A**) and contracted (**B**) virions. Scale bars = 100 nm. (**C**) Genome map of S13. The 227,647 bp genome includes 236 predicted open reading frames and has a GC content of 51.7%. Gene products are colour coded as proteins predicted to support nucleic acid metabolism (blue), virion structure and maturation (pink), lysis (red), jumbo phage nuclear shell (green), accessory proteins/phage proteins of unknown function (purple), and hypothetical proteins (gray). Scale is shown in base pairs (bp). Genome was circularized for visualization using Geneious Prime.

Phage vB_BgluM-SURPRISE13 (S13) was isolated from rhizosphere soil surrounding petunia (*Petunia exserta*) annual flowers collected in St. Albert, Alberta, Canada. Transmission electron micrographs revealed large virions with an average capsid diameter of 155.6 ± 4.7 nm, and contractile tails averaging 201.4 ± 4.3 nm in length, consistent with myovirus morphology (Fig. 1A and B). The genetic material of S13 consists of a 227,647 bp double-stranded DNA genome, with a GC content of 51.7%, 236 predicted protein-coding genes and no tRNAs (Fig. 1C). S13 is classified as a jumbo bacteriophage, with a genome exceeding 200 kb in length (55). Blastn analysis of the S13 genome revealed similarity to previously characterized jumbo phages RSL2 (56), RSF1 (56), FLC6 (11), and FLC8 (10) that are all members of the *Chiangmaivirus* genus within the *Chimalliviridae* family of phages. Comparative genome analysis of S13 and RSL2, RSF1, FLC6, or FLC8 produced average nucleotide identity (ANI) values of 84.22%, 83.98%, 84.53%, or 84.42%, respectively. Since these ANI values fall between 70% and 95% (57), this genomic comparison supports the assertion that S13 is a new member of the *Chiangmaivirus* genus of phages.

Despite intensifying interest in jumbo phages, many phage-encoded proteins remain functionally uncharacterized, including the products of core genes in certain jumbo phage groups (58). Of the 236 predicted coding sequences in the S13 genome, 61 were assigned putative functions based on protein sequence homology, and 175 were classified as hypothetical proteins (Fig. 1C; Table S3). Functional annotation of S13 revealed an arsenal of phage gene products with predicted roles in DNA metabolism and protection. A detailed list of these annotations can be found in Table S3. Briefly, S13 encodes nineteen predicted proteins involved in nucleic acid metabolism including DNA polymerases (gp3, gp194), an SH3 domain-containing protein (gp4), DNA helicases (gp217, gp128), an NAD-dependent DNA ligase (gp106), and numerous RNA polymerase subunits (gp219, gp233, gp40, gp41, gp51, gp218, and gp203) (Table S3). Genes encoding DNA replication, repair, transcription, and recombination are found in numerous jumbo phage genomes and may serve to increase phage autonomy by reducing reliance on host enzymes (59). Several S13-encoded proteins, including two ribonucleotide reductase subunits (gp119-120), dihydrofolate reductases (gp077-078), a thymidylate kinase (gp196), and a thymidylate synthase (gp167), are predicted to be involved in the synthesis of standard or modified pyrimidines, which may function to support phage replication or protect phage DNA from host restriction endonucleases (59, 60). Other functional annotations include fourteen virion structure and maturation proteins categorized as virion head maturation (gp126, gp36, gp26, and gp18), tail structure and assembly (gp30, gp42, gp66, and gp173), and virion structural proteins (gp31, gp60, gp69, gp102, gp180, and gp186). Annotated lysis proteins include a putative endolysin (gp92) and lytic transglycosylase (gp55).

S13 encodes 24 proteins representing accessory proteins or proteins with unknown function for bacteriophages. Notably, these include a cupin_2 family protein (gp70), radical SAM-domain containing proteins (gp76, gp71, gp79), 2OG-Fe(II) oxygenase family proteins (gp80, gp82), concanavalin A-like lectin/glucanases superfamily proteins (gp1, gp62-64, gp195), PhiKZ-like phage internal head family proteins (gp184-185), HAD-like proteins (gp114, gp162), N-acetyltransferase family proteins (gp21, gp23), and a QueC-like queuosine biosynthesis family protein (gp155) (Table S3). Several of these predicted proteins are found in other jumbo phage genomes and may represent structural components or may support phage replication in the presence of other phages or bacterial anti-phage defense systems (59).

Interestingly, S13 encodes a predicted tubulin nucleotide binding domain-like superfamily protein (gp11) that shares 80.77% identify (Evalue = 0.0) with the tubulin PhuZ protein of phage RSL2 and a hypothetical protein (gp235) that shares 92.13% identity with the predicted nuclear shell protein of RSF1 (Evalue = 0.0). Certain jumbo phages belonging to a group typified by *Pseudomonas* phage PhiKZ produce a nucleus-like compartment that has been shown to protect phage DNA from host-encoded CRISPR and restriction modification systems during phage infection of a host bacterial cell (61). Recently, PhiKZ was found to prevent phage resistance *in vitro* and increase the survival rate of *Pseudomonas-*infected *Galleria mellonella* more effectively than a non-nucleus forming phage, suggesting that phages producing these unique structures may be well suited for antimicrobial applications (62). Although not considered a core gene for nucleus-forming jumbo phages (58), some members of this group also encode the tubulin-like protein, PhuZ, that forms a cytoskeletal structure inside the host bacterium and can support phage production through various functions such as positioning and rotating the nuclear shell and translocating phage capsids toward the phage nuclear shell for DNA packaging (58, 63–65). Although the function of the S13-encoded nuclear shell (gp235) and tubulin nucleotide binding domain-like superfamily protein (gp11) have yet to be investigated, their presence contends that S13 shares a replication style with nucleus-forming phages. Our genetic analysis of S13 suggests that S13 is a novel jumbo phage with strong potential for therapeutic use and future biological discovery.

### Phage S13 uses flagella as a receptor and selects for flagellar mutations in *B. glumae*

**Fig 2:**
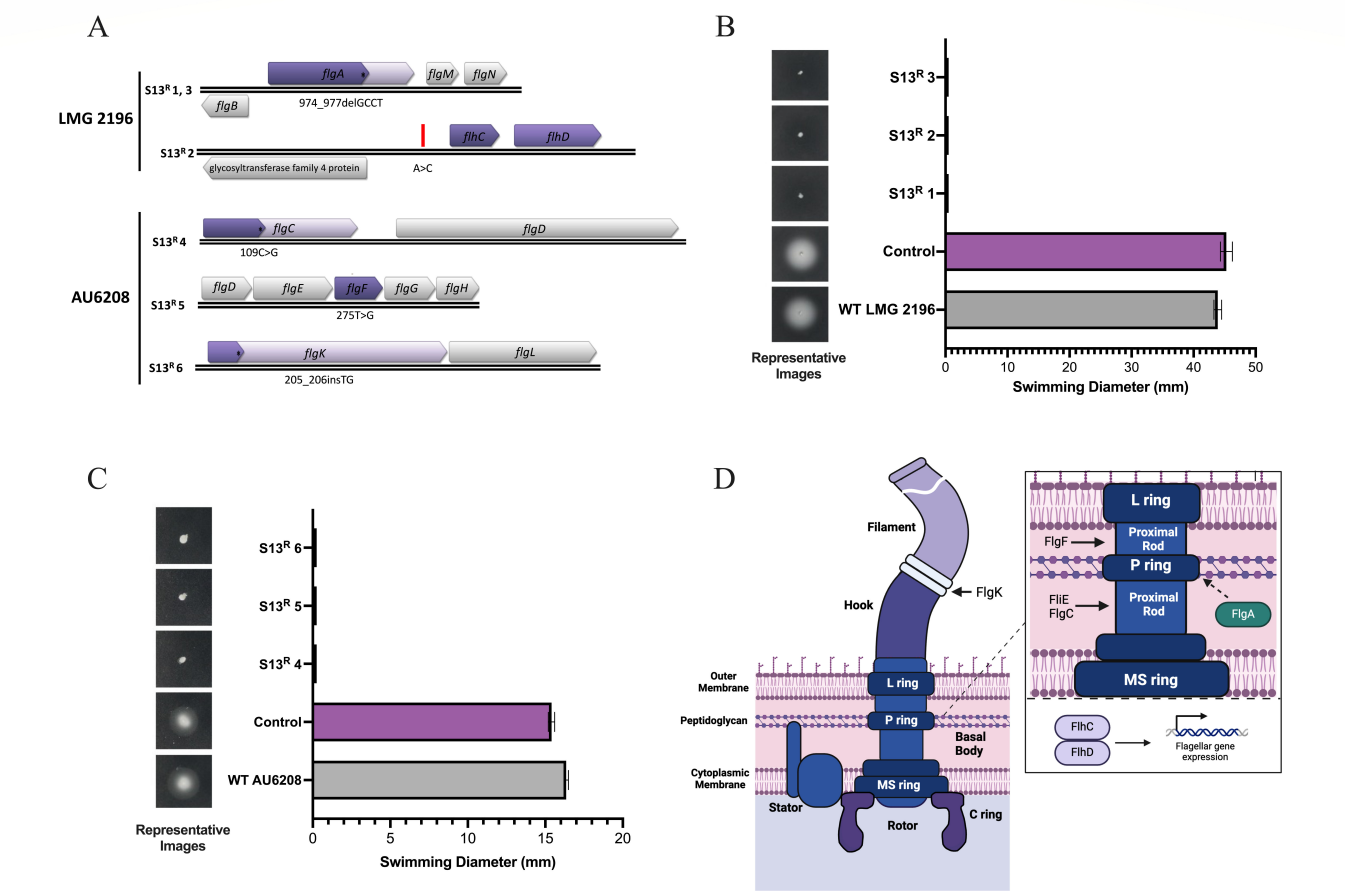
Mutational and phenotypic analysis of S13-resistant (S13^R^) *B. glumae* isolates. (**A**) Predicted flagella-associated mutations in S13^R^ *B. glumae* LMG 2196 and AU6208 isolates. Predicted protein truncations are shown in dark purple, with the WT protein shown in light purple. Intergenic mutations are shown in red. (**B and C**) Swimming motility of S13^R^ isolates, WT strains of (**B**) *B. glumae* LMG 2196 and (**C**) *B. glumae* AU6208, and control LMG 2196 or AU6208 strains evolved without S13. Error bars represent the standard error of the mean (SEM) for three biological replicates, with three technical replicates each. Representative swim zones for each strain are shown on the left of each plot. (**D**) Schematic of the predicted role of affected proteins in *B. glumae* flagellum structure, biosynthesis, and regulation adapted from (66). FlgC and FlgF are components of the flagellar proximal rod, FlgK comprises the putative hook-filament junction, and FlgA acts as a P-ring chaperone required for flagella biosynthesis. FlhC regulates the expression of flagella biosynthesis genes. Figure created with Biorender.com.

To characterize the cellular receptor recognized by S13, six *B. glumae* isolates were recovered from high MOI phage infections of strains LMG 2196 or AU6208 and sequenced to identify mutations in genes encoding cell surface-exposed receptors. Five of the six spontaneous S13-resistant (S13^R^) mutants contained predicted mutations in flagella-associated coding sequences or nearby intergenic regions (Fig. 2A) after analysis with Snippy/Breseq. Specifically, LMG 2196 isolates S13^R^ 1 and S13^R^ 3 contained a four bp deletion, (974_977delGCCT), causing a 178 amino acid truncation of FlgA. For LMG 2196 isolate S13^R^ 2, no coding-sequence mutations were identified; however, a single base pair substitution (A > C) was predicted 172 bp upstream of *flhC* (Fig. 2A). AU6208 isolate S13^R^ 4 contained a single base-pair (109C > G) substitution in *flgC,* introducing a premature stop codon and an 89 amino acid truncation of the peptide. AU6208 isolate S13^R^ 5 contained a predicted single base pair substitution (275T > G), resulting in a codon change (92 L > R) in FlgF. Finally, AU6208 isolate S13^R^ 6 showed a two bp insertion (205_206insTG) in *flgK,* causing a severe 596 amino acid truncation to the gene product. As expected, the WT strains and evolved *B. glumae* control isolates obtained from phage infection conditions without S13 retained swimming motility while S13^R^ mutants did not swim (Fig. 2B and C).

Flagella are intricate motility structures that are usually synthesized through the coordinated expression of >50 genes (66). Although *B. glumae* flagella biosynthesis has not been extensively characterized, flagellar biosynthesis has been thoroughly studied in *E. coli* and *Salmonella*, providing a basis for predicting the functional consequences of the mutations found here. In this work, several different mutations affected gene products predicted to be required for flagella structure and biosynthesis. Specifically, FlgC and FlgF represent core structural components of the flagellar proximal rod, which is a basal-body component that spans the space between the cytoplasmic and outer membrane (Fig. 2D). This structure transmits the torque generated by the motor complex to the extracellular hook and filament structures, enabling flagellar rotation (66, 67). Similarly, FlgK is a core structural component that comprises part of the hook-filament junction (Fig. 2D). This junction provides the point of attachment for the long, extracellular flagellar filament to the flagellar hook (66) and is required for motility in *Burkholderia pseudomallei* (68). Although not a structural component of the flagellum, FlgA is a chaperone protein involved in the assembly of the peptidoglycan-anchored P-ring (Fig. 2D) and is required for flagellar biosynthesis in several gram-negative pathogens such as *Salmonella enterica* and *Campylobacter jejuni* (69–71). In *B. glumae,* FlhDC comprises the master regulator of flagella biosynthesis, and FlhC mutants are non-flagellated (72). Although we did not confirm the importance of the intergenic region mutated in S13^R^ 2 to flagella biosynthesis, S13^R^ 2 lacked swimming motility, consistent with aberrant flagellar biosynthesis or function (Fig. 2B).

To verify that flagella-driven motility is required for S13 infection, we created isogenic flagella mutants in WT LMG 2196 and AU6208 strain backgrounds. Strains harboring clean deletions of *fliE,* a predicted proximal rod component (66), *flgC,* or *flgK* did not swim and were resistant to S13 in spotting assays, and genetic complementation restored both phage susceptibility and swimming motility in all strains (Table 1). Together, these results support the contention that S13 uses flagella as a receptor during phage infection.

**TABLE 1:** Swimming motility and S13 phage susceptibility of *B. glumae* flagella mutants and complemented strains

Bacterial strain	Swimming motility^*a*^	S13 spotting^*b*^
+pBBr1MCS		
WT LMG 2196	+	+
WT AU6208	+	+
LMG 2196 Δ*fliE*	−	−
AU6208 Δ*flgK*	−	−
AU6208 Δ*flgC*	−	−
+pBBr1MCS-complement		
LMG 2196 Δ*fliE*	+	+
AU6208 Δ*flgK*	+	+
AU6208 Δ*flgC*	+	+

^*a*^ +, swim zone detected after 24 h. −, no swim zone detected after 24 h.

^*b*^ +, phage activity at 10^8^ PFU/mL. −, no phage activity at 10^8^ PFU/mL.

Flagella-binding phages represent a morphologically diverse group of viruses that have been shown to use capsid or tail-associated receptor binding proteins to interact with the bacterial flagellum and are thought to traverse the rotating flagellar filament toward a secondary receptor on the bacterial cell surface (73–76).To date, the type IV pilus portal, lipopolysaccharide (LPS), lipooligosaccharide, and the antibiotic efflux-related complex, AcrABZ-TolC, are the only secondary receptors characterized for flagellotropic phages of Gram-negative bacteria (73, 74, 77, 78). Although the complete absence of S13 phage activity against constructed flagella-mutants points to the flagella as the sole receptor for S13 (Table 2), other researchers have theorized that synchronized binding to both the flagellum and a secondary receptor or flagella-mediated spatial positioning of the phage may be required for some flagella-binding phages (76). Therefore, to identify a potential secondary receptor used by S13, we isolated an additional 29 S13^R^ LMG 2196 mutants from three separate *in vitro* phage infections and looked for motile S13^R^ isolates. All mutants demonstrated swimming defects, suggesting that S13 frequently selects for non-motile bacterial populations *in vitro* (Fig. S1). In the absence of fully motile S13^R^ mutants, the existence of a possible secondary receptor in the S13 infection cycle is currently unknown.

**TABLE 2:** Host range of S13 phage against clinical and agricultural *Burkholderia* and *Ralstonia* isolates

*Burkholderia* species	Strain	EOP*^a^*	Swimming motility^*b*^	Source	Reference(s)
*B. cepacia*	CEP521	−	+	CF patient, Canada	CBCCRRR
	LMG 18821	−	+	CF patient, Australia	(79)
	LMG 18943	+	+	CF patient, USA	(80)
	Her1227	−	−		FdHRCBV
*B. cenocepacia*	H12424	3.3 × 10^−6^	+	Soil, USA	(81)
	715J	+++++	+	CF patient, USA	(82)
	D1	++	+	Soil, USA	(81)
	C5424	−	+	CF epidemic patient, Canada	(79, 83)
	C6433	−	−	CF epidemic patient, Canada	(79, 83, 84)
	C4455	+	+	CF epidemic patient, Canada	(85)
	CEP511	+	+	CF epidemic patient, Australia	(79, 86)
	R161	−	+	CF patient, Canada	(81)
	J2315	+	+	CF epidemic patient, UK	(79, 87, 88)
	R452	−	−	CF patient, Canada	(81)
	R750	−	+	CF patient, Canada	(81)
	R1619	0.96	+	CF patient, Canada	(81)
	CEP0868	0.23	+	CF patient, Argentina	(82)
	K56-2	+	+	CF epidemic patient, Canada	(79, 89)
	LMG 19240	++	+	Wheat soil, Australia	(90)
*B. multivorans*	M1512	+++	+	CF patient, Canada	(81)
	M1865	+++	+	CF patient, Canada	(81)
	R810	++++++	+	CF patient, Canada	(81)
	R1159	+++++	+	CF patient, Canada	(81)
	C5393	+++++	+	CF patient, Canada	(79, 85)
	C3430	+	+	CF patient, Canada	(85)
	C5568	−	+	CF patient, Canada	(85)
	LMG 13010	−	+	CF patient, Belgium	(79, 83, 85, 86, 91, 92)
*B. dolosa*	AU0158	+++	+	CF patient, USA	(80)
	CEP021	++++	+	CF patient, USA	(80)
	E12	+++	+	CF patient, UK	(80)
*B. gladioli*	CEP029	−	+	CGD patient, USA	(93)
	CEP071	6.2 × 10^−6^	+	Onion, Brazil	CBCCRRR
	CEP863	−	+	CF patient, Canada	CBCCRRR
	CEP950	+	+	CF patient, Canada	CBCCRRR
	D1170	++++++	+	CF patient, Canada	CBCCRRR
	CEP066	+	+	Onion, Canada	CBCCRRR
	R406	+	+	CF patient, Canada	(81)
*B. glumae*	LMG 2196	1.0	+	Rice, Japan	(94)
	AU6208	+++++	+	CGD patient, USA	(95, 96)
*B. lata*	St. 383	+	+	Soil, Trinidad	(97)
*B. ambifaria*	CEP996	++	+	CF patient, Australia	(80)
*B. pyrrocinia*	ATCC 39277	+	+	Soil, USA	(80)
	BC011	+	+	Water, USA	(80)
*Burkholderia* sp.	JS150	1.9 × 10^−6^	−	Soil, USA	(81)
*B. stabilis*	R450	−	+	CF patient, Canada	(81)
	LMG 14294	−	+	CF patient, Belgium	(79, 92)
*Ralstonia pickettii*	YH105	−	+	Soil, USA	(98)

^*a*^ Efficiency of plating (EOP) was calculated as the pfu/mL on each strain divided by the pfu/mL on the isolation host, *B. glumae* LMG 2196. In the absence of plaques, phage activity was scored as follows: −, no lysis detected; +, lysis at 10^8^ pfu/mL; ++, lysis at 10^7^ pfu/mL; +++, lysis at 10^6^ pfu/mL; ++++, lysis at 10^5^ pfu/mL; +++++, lysis at 10^4^ pfu/mL; ++++++, lysis at 10^3^ pfu/mL.

^*b*^ +, swim zone detected after 24 h at 30°C. −, no swim zone detected after 24 h at 30°C. Abbreviations: CBCCRRR, Canadian Burkholderia cepacia complex Research and Referral Repository; CF, cystic fibrosis; CGD, chronic granulomatous disease; FdHRCBV, Félix d'Hérelle Reference Center for Bacterial Viruses.

### Phage S13 demonstrates cross-species infective activity

The flagellin subunits composing the flagellar filament contain variable surface-exposed domains and may be post-translationally glycosylated or methylated to support diverse functions such as biofilm formation, host colonization, or immune system evasion by certain bacterial species (99–101). Since receptor compatibility impacts the phage host range, we tested the activity of S13 against a panel of clinical and agricultural isolates (Table 2), representing the broad diversity of *Burkholderia* species. S13 showed activity against nine different *Burkholderia* species including 22 strains isolated from human infections (Table 2). EOP analysis (28) revealed high phage productivity on *B. cenocepacia* strain R1619 (EOP = 0.96) and moderate phage productivity on *B. cenocepacia* CEP0868 (EOP = 0.23). Despite the absence of discernable plaques, S13 was highly or moderately active in spotting assays against 11 additional strains: five strains of *Burkholderia multivorans,* three strains of *Burkholderia dolosa,* one strain of *B. glumae,* one strain of *Burkholderia gladioli,* and one strain of *B. cenocepacia*. (Table 2). Weak activity was seen against 15 other strains across several species (Table 2). Generally, plaque formation indicates successful phage replication, whereas clearing without plaque formation may be due to abortive infection or lysis from without, which involves adhesion to surface receptors but does not result in the production of new phage progeny (28, 102). However, plaque formation may be impeded by several factors including phage latent period and burst size (103). In our study, we observed poor diffusion of the large phage particles through solid media during spotting assays, which may contribute to the lack of plaque formation observed on certain strains. Taken together, our results show that S13 is broadly active against several *Burkholderia* species that produce compatible receptors for S13 binding.

Cross-species activity has also been reported for related *B. glumae* jumbo phages FLC6 and FLC8, which are both active against some strains of *Burkholderia plantarii* (10, 11). Although the receptors used by these phages remain unknown, this broad activity may reflect the unique biology of large-genome phages, whose metabolism-related proteins and nucleotide modification systems may reduce intracellular barriers to phage infection (59). On the other hand, cross-species activity is seen for some, but not all, flagellum-binding phages (76). For example, jumbo *Caulobacter crescentus* phage, CbK, demonstrates some activity against the related species, *Caulobacter segnis* (104), whereas *Agrobacterium* phage 7-7-1 only infects *Agrobacterium* sp. H13-3 and is not active against other related *Agrobacterium* or *Rhizobium* species (105). In our study, no significant phage activity was seen against non-swimming strains (Table 2). Furthermore, phage activity was not detected during spotting assays of S13 on a filament-deficient (Δ*fliC*) mutant strain of *Burkholderia cenocepacia* K56-2, supporting that flagella is likely required for host recognition across *Burkholderia* species (data not shown). However, some motile strains were not susceptible to S13 (Table 2), indicating that motility is not the only requirement for phage-host compatibility, and factors such as flagellin variation, differences in intracellular barriers to phage infection, or variation of potential secondary receptor structures may play a role in S13 activity. In the future, an in-depth characterization of the flagellins encoded by susceptible strains and identification of S13 receptor binding proteins may shed light on additional potential hosts for S13.

### Phage S13 lytic activity is impacted by temperature

**Fig 3:**
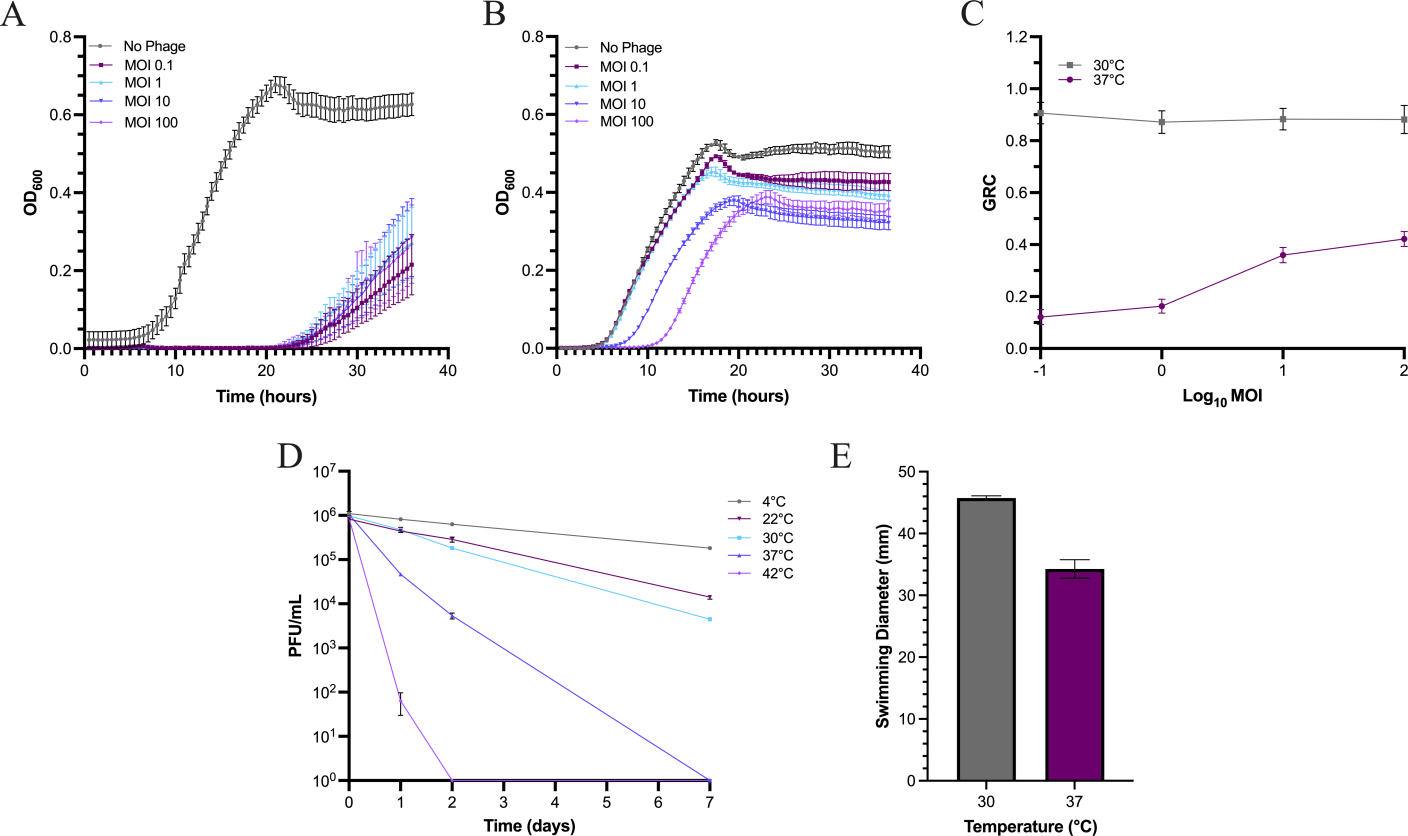
Temperature-dependent activity of S13 phage against *B. glumae* LMG 2196. (A and B). *In vitro* growth reduction of *B. glumae* LMG 2196 treated with S13 at various MOI at (**A**) 30°C or (**B**) 37°C. Data points show the average OD_600_ of four biological replicates with error = standard error of the mean (SEM). (**C**) Growth reduction coefficient (GRC) of S13 at 30°C or 37°C across MOIs. (**D**) Temperature stability of S13 lysates incubated at 4, 22 (room temperature), 30, 37, or 42°C across time. Data show the average of three replicate experiments with error = SEM. (**E**) Swimming motility of *B. glumae* LMG 2196 at 30°C and 37°C. Data show the average measurements from three biological replicates with three technical replicates each. Error = SEM.

Given the broad activity of S13 against *B. glumae* and clinical *Burkholderia* pathogens (Table 2), S13 may be useful for both agricultural and clinical phage applications. Therefore, we evaluated the *in vitro* activity of S13 at representative environmental (30°C) and human physiological (37°C) temperatures. Liquid infections of the isolation host *B. glumae* LMG 2196 with S13 were conducted at MOIs ranging from 0.1 to 100. At 30°C, S13 prevented bacterial growth for 24 h (Fig. 3A). However, at 37°C, S13 was less effective, with bacterial outgrowth occurring after only 5.5 h at low MOIs, 7.5 h at an MOI of 10, and 11 h an MOI of 100 (Fig. 3B). Quantification of this activity using the growth reduction coefficient (GRC) (31) indicates that S13 activity remained high across all MOIs at 30°C, with values ranging between 0.86 ± 0.03 and 0.91 ± 0.04 (mean ± SEM) of a maximum of 1.0. At 37°C, GRC values were generally lower but increased from 0.12 ± 0.03 to 0.42 ± 0.04 (mean ± SEM) with increasing MOI (Fig. 2C).

Although this comparative reduction in activity at 37°C may be partially due to reduced bacterial growth at this temperature (Fig. 3A and B), the MOI dependence observed at 37°C but not at 30°C led us to hypothesize that heat inactivation of virions reduces S13 efficacy. Thus, we monitored phage stability over time at storage temperature (4°C) or at selected treatment-relevant temperatures between 22°C and 42°C (Fig. 3D). Lysates incubated at 30°C or lower remained stable after 48 h, with minimal reductions in PFU/mL (Fig. 3D), suggesting good short-term stability. Conversely, after 48 h, active phage particles decreased by ~200-fold of the starting PFU/mL at 37°C and were completely deactivated at 42°C (Fig. 3D), suggesting that S13 virions are heat labile. After 7 days, lysates incubated at 4°C, 22°C, and 30°C decreased by 6-fold, 60-fold, and 200-fold of the starting PFU/mL, respectively (Fig. 3D), indicating that S13 stability decreases over time with increasing temperature. Although the stability of related jumbo phage, FLC8, at storage temperatures between 4°C and −80°C has been investigated (10), the stability of FLC8 and other *Chiangmaiviruses* at representative field temperatures remains unknown. Our results suggest that heat instability likely contributes to reduced S13 activity at 37°C.

*B. glumae* LMG 2196 exhibited decreased swimming motility at 37°C in comparison to 30°C (Fig. 3E). Temperature is a known regulator of flagella biosynthesis in *B. glumae,* and strain BGR1 has been shown to produce fewer flagella at 37°C compared with 28°C (106). Since S13 requires flagella for infection, reduced flagellation at 37°C could also contribute to reduced phage activity at this temperature. Although most strains in our host range panel produced larger swim zones at 37°C in comparison to 30°C, smaller swim zones at 37°C were seen for several strains including *Burkholderia stabilis* R450, *B. dolosa* E12*, B. gladioli* R406, *B. gladioli* CEP0950*, B. cenocepacia* R161, and *R. pickettii* YH105 (data not shown). These findings posit that temperature-dependent host factors such as reduced growth rate or receptor expression could present an obstacle to phage infection of certain strains across several *Burkholderia* species.

Temperature-dependent activity has been observed for some *Burkholderia* phages such as *B. cenocepacia* phage JC1, which displays greater lytic activity at 37°C than at 30°C (22), and phage ΦBp-AMP1, which lyses liquid cultures of *B. thailandensis* more effectively at 37°C than at 25°C (107). Further investigation of ΦBp-AMP1 showed increased lysogeny at 25°C, suggesting that temperature plays a role in the switch from lysogenic to lytic life cycle in some lysogeny-capable phages (107). In contrast to those studies, our data suggest that elevated temperatures may reduce S13 lytic phage activity through reduced virion stability or host cell changes. Although 28°C is considered the optimal daytime temperature for rice cultivation (108), *B. glumae* tolerates temperatures above 40°C, and disease outbreaks have been associated with warm, humid conditions (1, 2, 109). Considering the temperature-dependent activity of S13 observed in our work, strategies such as timed application of phages during cool periods or seed treatment under controlled conditions (110) may maximize the usefulness of S13 for controlling *B. glumae* infections.

### Rice seedling rot is controlled *in planta* by S13

**Fig 4:**
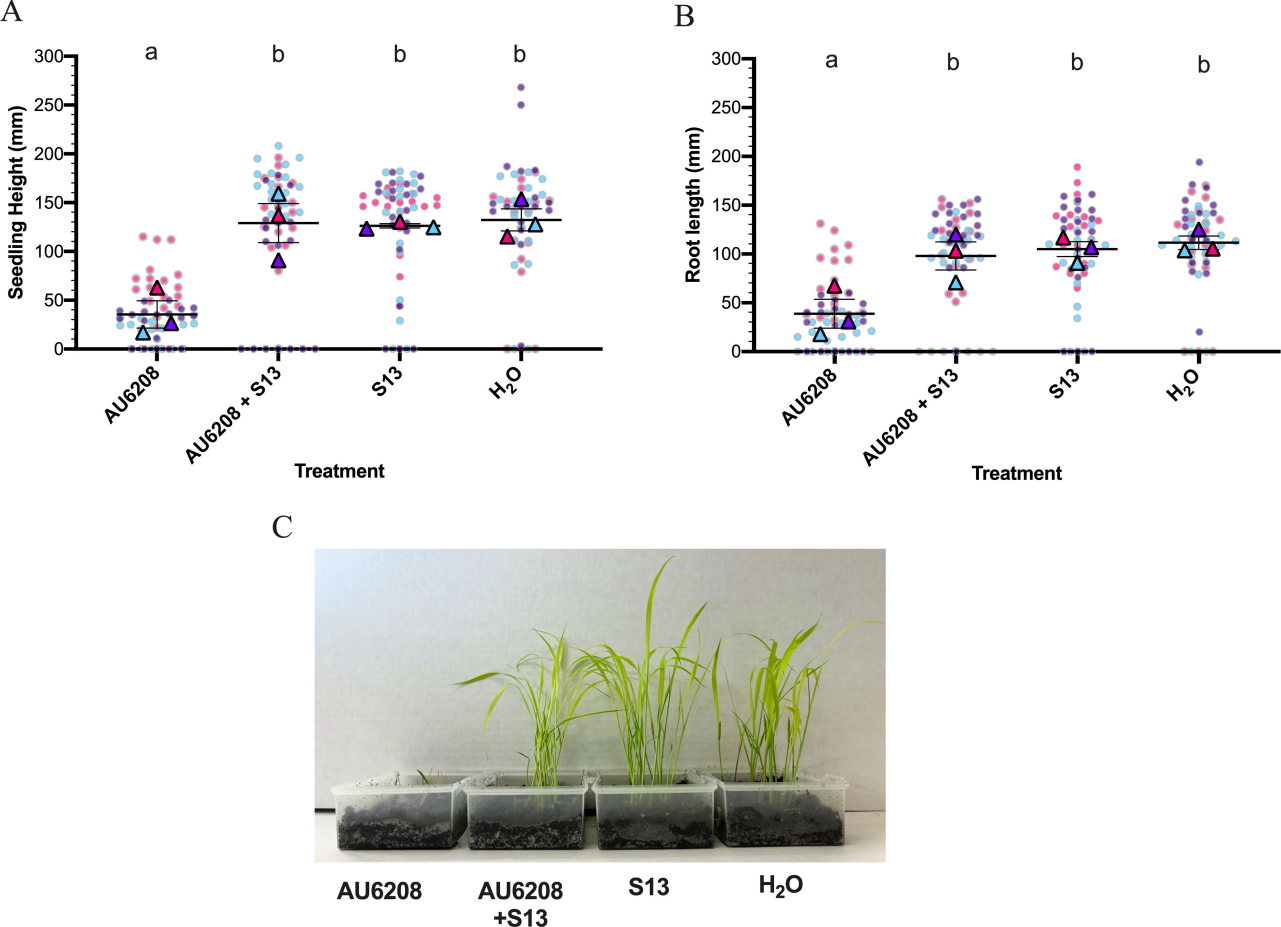
S13 phage-mediated suppression of rice seedling rot. (A and B). Seedling height (**A**) and root length (**B**) of AU6208-inoculated, or uninoculated *Oryza sativa* “zerawchanica” seeds germinated with or without S13. Measurements were taken 10 days after growth at 30°C on pre-sterilized soil. Lines represent the average values obtained from three replicate experiments, with error bars showing the standard error of the mean (SEM). Results for each individual replicate are shown as colored triangles and represent the average of measurements from 18 to 20 individual seedlings (colored circles). Treatments groups with the same letter do not differ significantly as determined by a nested one-way ANOVA with Tukey’s multiple comparisons test at *P* = 0.05. (**C**) Representative image of seedlings after 10 days of growth.

After exploring the *in vitro* activity of S13 at optimal rice-growing temperatures, we investigated the *in-planta* efficacy of S13 against rice seedling rot. In preliminary tests, the S13 isolation host *B. glumae* LMG 2196 did not induce rot symptoms in seedlings, even at high cell concentrations of 10^8^ CFU/mL (data not shown). Therefore, we used *B. glumae* AU6208, a clinical isolate known to be virulent in rice plants when inoculated during the flowering stage (95) for our experiments. Rice seeds were inoculated with *B. glumae* AU6208 and subsequently germinated in the presence or absence of S13 before sowing on commercial soil. After 10 days, seedlings were assessed for stem and root truncation (54) (Fig. 4A through C). Inoculation with AU6208 produced plants with severely truncated stems (*P* = 0.0043) and shorter roots (*P* = 0.0093) than water-only controls, whereas germination of infected seeds with S13 restored both seedling height (*P* = 0.0053) and root length (*P* = 0.028). Moreover, uninfected seeds germinated with S13 grew to similar levels as water-only controls, suggesting that S13 does not cause adverse effects on rice seedlings (Fig. 4A through C). Our results indicate that seed treatment with S13 is a safe and efficacious method for reducing *B. glumae* seedling disease during the early stages of rice development.

Seedling diseases impact rice cultivation in nurseries and are caused by a diverse array of pathogens (111). Successful rice seedling rot suppression has been achieved using S13-related jumbo phages FLC6 (11), FLC8 (10), and a genetically distinct jumbo phage, FLC9 (10). Combined with the broad activity of S13 against several *Burkholderia* species, including *B. gladioli* (Table 2), a related *Burkholderia* pathogen capable of infecting rice (109), our results highlight S13 as a potentially useful new control agent for seed diseases caused by *B. glumae* and related pathogens.

### Phage S13 steers *B. glumae* populations toward reduced virulence in rice seedlings

**Fig 5:**
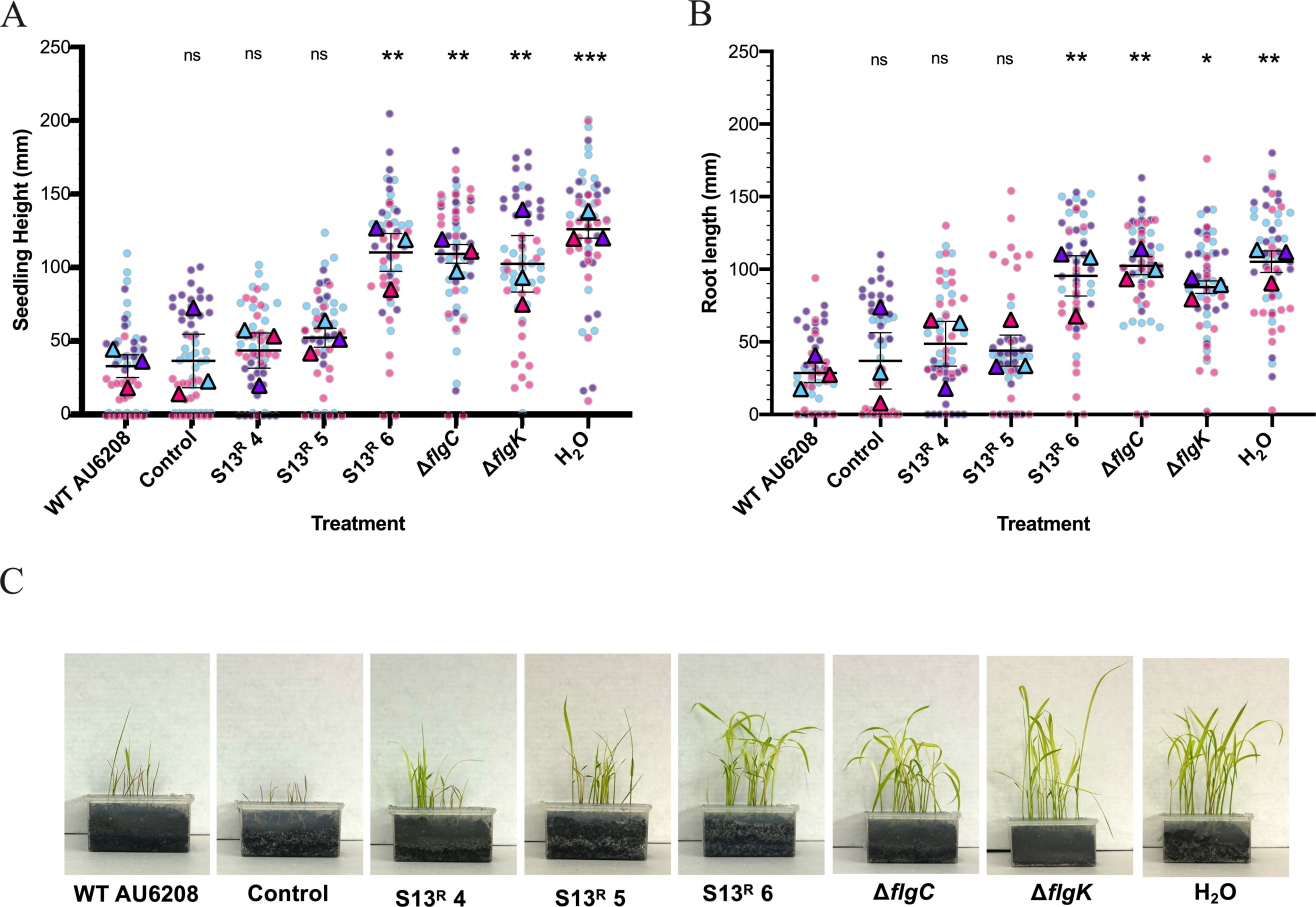
Virulence of swimming-deficient *B. glumae* AU6208 strains in rice seedlings. (**A and B**) Seedling height (**A**) and root length (**B**) of *Oryza sativa* “Zerawchanica” seeds inoculated with *B. glumae* AU6208 flagella mutants. Measurements were taken 9 days after growth at 30°C on pre-sterilized soil. Lines represent the average values obtained from three replicate experiments, with error bars showing the standard error of the mean (SEM). Colored triangles show the results for each replicate experiment and represent the average values from 18 to 20 individual seedlings. Measurements from each individual seedling are shown as colored circles. Significant differences in comparison to WT AU6208 were determined using a nested one-way ANOVA with Dunnett’s post-test (ns, not significant; *, *P* < 0.03; **, *P* < 0.01; ***, *P* < 0.001). (**C**) Representative image of seedling growth after 9 days.

Flagella are important to the virulence of several *Burkholderia* pathogens including *B. glumae, B. pseudomallei,* and *B. cepacia* (72, 112, 113). Therefore, we sought to determine if non-motile S13^R^ *B. glumae* isolates showed altered virulence in rice seedlings (Fig. 5). Seedlings inoculated with WT AU6208 or the evolved AU6208 control strain grew poorly in comparison to water-only controls (*P* = 0.0003*)*, whereas inoculation with S13^R^ 6 produced taller seedlings (*P* = 0.0021*)* with longer roots (*P* = 0.0050) than WT AU6208, similar to the water-only controls (Fig. 5A and B). Similarly, seedlings inoculated with clean deletion strains of Δ*flgk* and Δ*flgC,* lacking structural components of the hook-filament junction and proximal rod, respectively, were taller (*P* = 0.0053; *P* = 0.0024) and grew longer roots (*P* = 0.013; *P* = 0.0021) than WT controls, indicating that these flagella structures are necessary for full virulence in rice seedlings. Together, these findings suggest that receptor mutations acquired to evade S13 predation can reduce the virulence of *B. glumae* in rice seedlings.

Despite the observed decrease in virulence for clean-deletion proximal rod mutant, Δ*flgC,* infection of seedlings with S13^R^ 4 and S13^R^ 5, which contain single base-pair mutations in proximal-rod encoding genes *flgC* and *flgF,* respectively (Fig. 2A), remained capable of reducing seedling growth similar to WT AU6208 (Fig. 5A and B). During *in vitro* tests, S13^R^ 4, S13^R^ 5, and Δ*flgC* demonstrated consistent growth patterns and reached similar maximum OD_600_ values in LB media (Fig. S2A). Additionally, increased pellicle formation was observed for all three proximal rod mutants in static cultures, and further investigation showed increased adherent biofilm formation for these strains in comparison to WT AU6208 (Fig. S2B and C).

Given that S13^R^ 4 and 5 demonstrated similar phenotypes to Δ*flgC in vitro* (Fig. S2)*,* but not *in planta* (Fig. 5)*,* we suspected that reversion or suppression of the single base-pair mutations (Fig. 2A) in these mutants may occur under rice germination or growth conditions, causing the retained virulence of these strains. To our knowledge, no examples of mutation suppression, hypermutable regions, or reversion of spontaneous flagella mutations have been reported for *Burkholderia* species. However, in a recent study, in-frame deletions or single codon changes in *fliE* mutants of *Salmonella enterica* were shown to be suppressed by single codon substitutions in proximal rod proteins *FlgC* or *FlgB* (114). To explore the possibility of phenotypic reversion in our study, S13^R^ 4 and S13^R^ 5 cultures were incubated in milliQ H_2_O for 48 h to replicate seed germination conditions, followed by inoculation into swim agar (data not shown). In these tests, motile variants of S13^R^ 5 were detected after 8 days of growth in swim agar. No motile variants of S13^R^ 4 were identified; however, seedling growth conditions may present additional selective pressures favoring reversion to a motile phenotype *in planta.*

Although flagella-binding phages have been hypothesized to act as anti-virulence agents by selecting for non-motile and thus avirulent populations (76), few examples of this process have been reported (115). Overall, the virulence reductions observed for phage-resistant mutants Δ*flgK* and Δ*flgC* (Fig. 5) support the contention that flagella are required for *B. glumae* disease progression in rice seedlings and may be exploitable targets for phage biocontrol strategies aimed at steering *B. glumae* toward reduced virulence. Furthermore, the attenuation of S13^R^ 6 in our work (Fig. 5) indicates that S13 mutational pressure directly reduces *B. glumae* virulence through receptor loss. These results suggest that S13 is a promising candidate for antimicrobial and anti-virulence phage applications. However, the phenotypic variation observed for different spontaneous S13^R^ mutants *in planta* brings up the complexities of the rice-bacterial infection interaction. Although flagella are necessary for *B. glumae* virulence in rice (72)*,* bacterial flagellins are known antigens recognized by system-triggering pattern recognition receptors (PRRs) on plant cell surfaces, and many plant-associated bacteria employ strategies to downregulate or mask flagellins to avoid plant immune detection (116). Flagellin-triggered immunity has been observed in rice, as transgenic seedlings modified to express *Acidovorax avenae* flagellins showed increased immune system activation (117). As such, selective pressures related to the dual role of flagella in virulence and antigenicity may complicate the interaction between motile plant pathogens of rice and flagellum-binding phages. Therefore, the frequency and impact of phage-driven receptor loss *in planta* requires additional investigation.

In summary, we have discovered a new *B. glumae*-specific bacteriophage S13 that exhibits an extremely broad host range due to its ability to utilize the *B. glumae* flagella as its cellular receptor. Not only does such an attachment mechanism serve to provide effective phage DNA access to the cell, but the mutational loss of the flagella to prevent phage infection places the cell in an avirulent state. Furthermore, we reveal that S13 treatment of *B. glumae* in a rice seedling infection model, even without the use of a phage cocktail to prevent phage resistance from evolving, is highly effective at providing protection from bacterial disease and significantly enhancing rice plant growth.

## Data Availability

The complete S13 genome was deposited to GenBank (accession: PP856017).

## SUPPLEMENTAL MATERIAL

Figure S1 mbio.02814-24-s0001.pdf Swimming motility of additional fully and partially S13-resistant *B. glumae* LMG 2196 isolates.

Figure S2 mbio.02814-24-s0002.pdf Growth and biofilm formation of constructed and spontaneous S13-resistant *B. glumae* AU6208 flagella mutants.

Table S1 mbio.02814-24-s0003.docx *B. glumae* strains and plasmids used in this study.

Table S2 mbio.02814-24-s0004.pdf Primers used in this study.

Table S3 mbio.02814-24-s0005.pdf Phage S13 genome annotations obtained by protein sequence/domain homology analysis.

ASM does not own the copyrights to Supplemental Material that may be linked to, or accessed through, an article. The authors have granted ASM a non-exclusive, world-wide license to publish the Supplemental Material files. Please contact the corresponding author directly for reuse.
