# Supplementary material for: A flagella-dependent Burkholderia jumbo phage controls rice seedling rot and steers Burkholderia glumae toward reduced virulence in rice seedlings
Source: mBio. 2025 Jan 27;16(3):e02814-24. doi: 10.1128/mbio.02814-24 (PMC11898562; doi:10.1128/mbio.02814-24)
Supplement: Figure S2 — Growth and biofilm formation of constructed and spontaneous S13-resistant B. glumae AU6208 flagella mutants. [file mbio.02814-24-s0002.pdf]

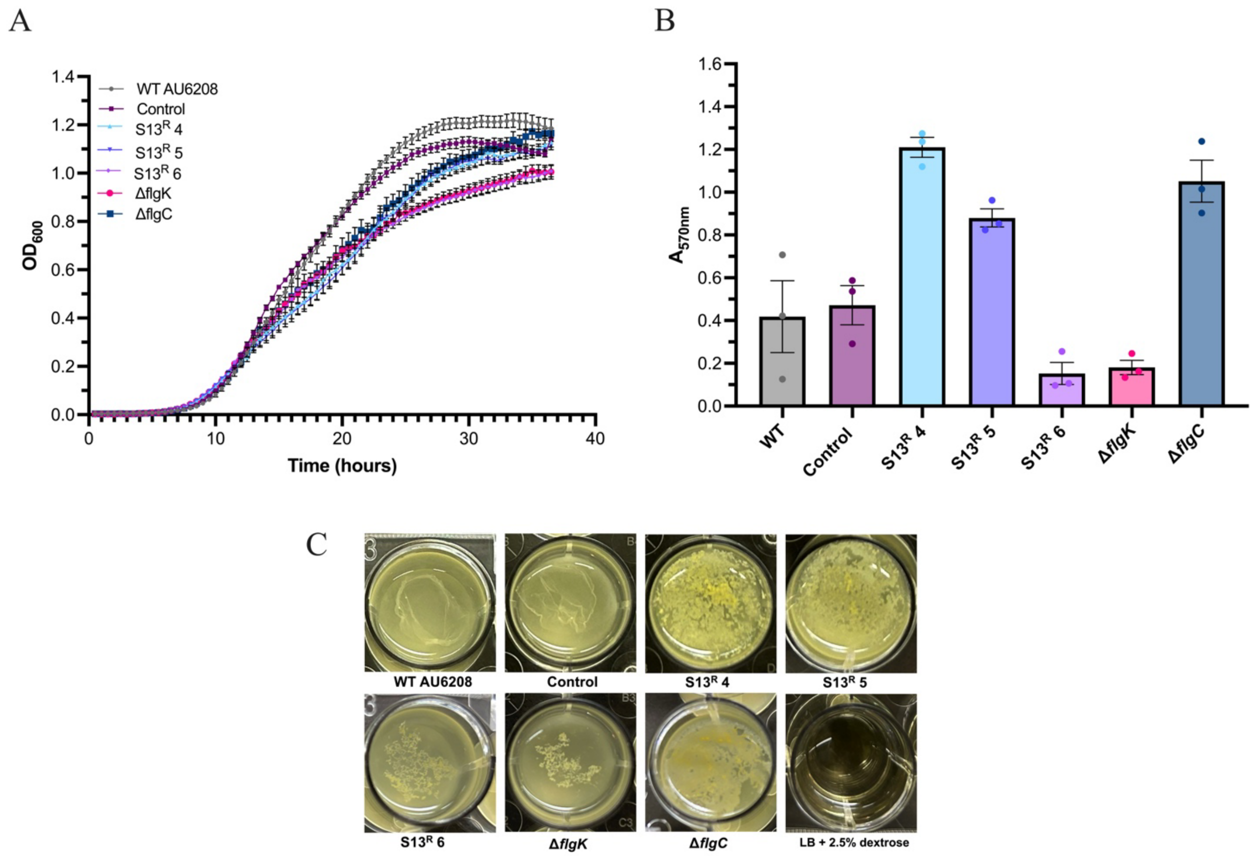

Figure S2: Growth and biofilm formation of constructed and spontaneous S13-resistant ($S13^R$) *B. glumae* AU6208 flagella mutants. **A.** Growth curves of AU6208 strains in full strength Lennox broth (LB). Strains $S13^R$ 4, & $S13^R$ 5 which have predicted mutations proximal-rod structural genes demonstrate similar growth to constructed proximal rod mutant, Δ*flgC,* while $S13^R$ 6, which harbors a mutation in the hook-filament junction gene, *flgK* grows similarly to Δ*flgK*. Error bars depict standard error of the mean (SEM). **B-C.** Biofilm formation of *B. glumae* AU6208 strains in LB + 2.5% dextrose after 48 hours at 30 °C. (B) Adherent biofilms stained with 1% Crystal violet and quantified at 570nm. Error bars represent SEM, while individual points show the measurements from 3 biological replicates. (C) Representative image of pellicles formed at the air-surface interface of wells during biofilm experiments. All experiments were conducted in biological triplicate with two technical replicates each.
