# Supplementary material for: A flagella-dependent Burkholderia jumbo phage controls rice seedling rot and steers Burkholderia glumae toward reduced virulence in rice seedlings
Source: mBio. 2025 Jan 27;16(3):e02814-24. doi: 10.1128/mbio.02814-24 (PMC11898562; doi:10.1128/mbio.02814-24)
Supplement: Table S1 — B. glumae strains and plasmids used in this study. [file mbio.02814-24-s0003.docx]

Table S1. *B. glumae* strains and plasmids used in this study

| **Bacterial Strain** | **Description** | **Source** |
| --- | --- | --- |
| LMG 2196 | Wildtype Strain (ATCC 33617) |  |
| LMG 2196 Δ*fliE* | Clean deletion of *fliE* in LMG 2196 | This study |
| AU6208 | Wildtype strain | (96) |
| AU6208 Δ*flgK* | Clean deletion of *flgK* in AU6208 | This study |
| AU6208 Δ*flgC* | Clean deletion of *flgC* in. AU6208 | This study |
| **Plasmids** |  |  |
| pBBR1MCS | Broad-host range expression vector, Cm^R^ | (50) |
| pGPI-*SceI* | Mutagenesis suicide plasmid, Tp^R^ | (49) |
| pDAI-*SceI* | Broad-host range vector with *I-SceI* nuclease gene, Tc^R^ | (49) |
| pLMG2196*fliE* | pBBR1MCS carrying LMG 2196 *fliE*, Cm^R^ | This study |
| pAU6208*flgK* | pBBR1MCS carrying AU6208 *flgK*, Cm^R^ | This study |
| pAU6208*flgC* | pBBR1MCS carrying AU6208 *flgC*, Cm^R^ | This study |
