# Supplementary material for: A flagella-dependent Burkholderia jumbo phage controls rice seedling rot and steers Burkholderia glumae toward reduced virulence in rice seedlings
Source: mBio. 2025 Jan 27;16(3):e02814-24. doi: 10.1128/mbio.02814-24 (PMC11898562; doi:10.1128/mbio.02814-24)
Supplement: Table S2 — Primers used in this study. [file mbio.02814-24-s0004.pdf]

Table S2. Primers used in this study.

**Primer Name**	**Sequence (5'-3')***
**Strain Construction Primers**	
fliE Up	TACT**TCTAGA**GTCTCGCTACTGGTCCTTAC
fliE OE Up	GGAGATCCCATGACTATGTCGGTCTGACCCCTA
fliE Down	AATA**GGTACC**GTAGAGATCGACGAACACC
fliE OE Down	GGGTCAGACCGACATAGTCATGGGATCTCCGCA
flgK Up	AGTA**TCTAGA**CCCACAGGCAGGCCATCAG
flgK OE Up	TCGTCAGTTGAAGATAAGGTTGCTCATGGGATGC
flgK Down	TACT**GGTACC**GTTCTGCATCTTGGTCAGCG
flgK OE Down	CCCATGAGCAACCTTATCTTCAACTGACGACGGAA
flgC Up	TACT**TCTAGA**GCTGTACGAAGCGAGGCT
flgC OE Up	ACACGGCACTCAGGTCGAAGGCATGGAAATTCCTCG
flgC Down	TCAC**GGTACC**ACCCATGTTGGTCTCCGA
flgC OE Down	ATTTCCATGCCTTCGACCTGAGTGCCGTGTCGT
**Complementation Primers:**	
fliE CompF	TACT**TCTAGA**CGTTATCGGTAAGGATGGGG
fliE CompR	ATTG**CTCGAG**GAACAGAGCCGAGTTTTCCC
flgK CompF	TACT**GGTACC**GACGCCGCCGTTATACCG
flgK CompR	CAGC**TCTAGA**TCATCAGCTCGATGTTCTGG
flgC CompF	ACTC**GGTACC**CACGCCGCCATTCTTTCAG
flgC CompR	CTCA**TCTAGA**GCTCATCTCCGGGTTGCCTT
**S13 Genome Assembly Verification Primers**	
S13-1F	CAGGTGAAGGCGAGATCATC
S13-1R	ATTTCGTCGTCAGGCAGAAC
S13-2F	CGAAAGCAAAGAGGTCAAAG
S13-2R	TGAAATGGATAAGTCGGACC
S13-3F	GTTCTCCAGTACATTCTTTCAG
S13-3R	AACAGTGGTGAGTTTGGTG
S13-4F	CATCTCCCCGAACTGAGGAA
S13-4R	GAAACTGAACCGCCTGTACG
S13-5F	GCCGACAATTAGAGAGACAT
S13-5R	GAAGATGATCTCGGACGAAA
S13-6F	GCAAGATGAATCAGAGCGAACT
S13-6R	TTACAAGAATGGCGCACAGG
S13-7F	ACGTTGGGAATCTAGCATACAC
S13-7R	ACTGATCTGATTCGACATGGAT

* Restriction enzyme recognition sites for *XhoI*, *XbaI*, and *KpnI* are bolded and underlined
