# Supplementary material for: A flagella-dependent Burkholderia jumbo phage controls rice seedling rot and steers Burkholderia glumae toward reduced virulence in rice seedlings
Source: mBio. 2025 Jan 27;16(3):e02814-24. doi: 10.1128/mbio.02814-24 (PMC11898562; doi:10.1128/mbio.02814-24)
Supplement: Table S3 — Phage S13 genome annotations obtained by protein sequence/domain homology analysis. [file mbio.02814-24-s0005.pdf]

Table S3. Phage S13 genome annotations obtained by protein sequence / domain homology analysis.

Name	Min (bp)	Max (bp)	Direction	Predicted Functional Category	Product	Annotation Evidence			
						Similar Protein	E Value	% Identity	Protein Motifs
SURPRISE13_001	22	726	-	accessory/unknown function	concanavalin A-like lectin/glucanases superfamily protein	BDD79519.1	3.00E-105	70.42%	SUPFAM:SSF49899:Concanavalin A-like lectins/glucanases
SURPRISE13_002	726	1397	-	hypothetical protein	hypothetical protein	BCM95038.1	6.00E-116	76.74%	
SURPRISE13_003	1472	3613	-	nucleic acid metabolism	DNA polymerase	YP_009212852.1	0	90.32%	PFAM:PF20286:divDNApol
SURPRISE13_004	3724	4152	-	nucleic acid metabolism	SH3 domain-containing family protein	BCM95040.1	5.00E-85	88.41%	PFAM:PF20287:SH3DP
SURPRISE13_005	4165	4542	-	hypothetical protein	hypothetical protein	BCM95041.1	3.00E-70	85.37%	
SURPRISE13_006	4527	4958	-	hypothetical protein	hypothetical protein	YP_009207810.1	1.00E-38	48.68%	
SURPRISE13_007	5014	5370	-	hypothetical protein	hypothetical protein	YP_009207811.2	4.00E-75	91.53%	
SURPRISE13_008	5370	5795	-	hypothetical protein	hypothetical protein	BCM95044.1	3.00E-85	83.69%	
SURPRISE13_009	5917	6342	-	hypothetical protein	hypothetical protein	YP_009212858.1	8.00E-85	85.82%	
SURPRISE13_010	6329	6904	-	hypothetical protein	hypothetical protein	BCM95046.1	1.00E-125	90.05%	
SURPRISE13_011	7101	8117	-	phage nuclear shell	tubulin nucleotide-binding domain-like superfamily protein	YP_009212860.1	0	80.77%%	SUPFAM:SSF52490:Tubulin_nucleotide-binding_domain-like
SURPRISE13_012	8227	9039	+	hypothetical protein	hypothetical protein	YP_009212861.1	0	90.00%	
SURPRISE13_013	9097	9426	-	hypothetical protein	hypothetical protein	YP_009207817.2	3.00E-31	52.17%	
SURPRISE13_014	9537	9827	-	hypothetical protein	hypothetical protein	YP_009212863.1	7.00E-32	64.52%	
SURPRISE13_015	9874	10014	-	hypothetical protein	hypothetical protein	YP_009241388.1	3.00E-10	65.22%	
SURPRISE13_016	10059	10523	-	hypothetical protein	hypothetical protein	BCM95052.1	4.00E-66	67.11%	
SURPRISE13_017	10547	10741	-	hypothetical protein	hypothetical protein	BCM95053.1	3.00E-29	85.25%	
SURPRISE13_018	10873	12186	-	virion structure and maturation	AAA-family ATPase	BCM95054.1	0	66.89%	PFAM:PF08740:BCS1_N; PFAM:PF00004:AAA
SURPRISE13_019	12290	13054	-	hypothetical protein	hypothetical protein	BDD79301.1	6.00E-121	77.33%	
SURPRISE13_020	13445	14092	+	hypothetical protein	hypothetical protein	BCM95056.1	6.00E-128	82.79%	
SURPRISE13_021	14104	14535	+	accessory/unknown function	N-acetyltransferase domain-containing protein	YP_009212869.1	1.00E-83	96.03%	CDD:cd04301:NAT_SF
SURPRISE13_022	14545	15792	+	hypothetical protein	hypothetical protein	YP_009212870.1	0	98.31%	
SURPRISE13_023	15797	16216	+	accessory/unknown function	N-acetyltransferase family protein	BAU71403.1	9.00E-55	59.85%	PFAM:PF00583:Acetyltransf_1
SURPRISE13_024	16247	16426	+	hypothetical protein	hypothetical protein	YP_009207828.1	2.00E-30	89.83%	
SURPRISE13_025	16463	16645	+	hypothetical protein	hypothetical protein	BCM95060.1	1.00E-27	91.23%	
SURPRISE13_026	16688	18868	-	virion structure and maturation	terminase large subunit	YP_009212872.1	0	89.94%	
SURPRISE13_027	18941	20659	-	hypothetical protein	hypothetical protein	BCM95062.1	0	91.08%	
SURPRISE13_028	20672	23272	-	hypothetical protein	hypothetical protein	YP_009212874.1	0	88.22%	
SURPRISE13_029	23341	24240	-	hypothetical protein	hypothetical protein	YP_009207833.1	0	90.64%	
SURPRISE13_030	24351	26456	+	virion structure and maturation	tail sheath protein	BCM95065.1	0	89.05%	PFAM:PF20961:phiKZ_gp29PR
SURPRISE13_031	26473	27339	+	virion structure and maturation	virion structural protein	YP_009212877.1	0	98.61%	
SURPRISE13_032	27411	28709	-	hypothetical protein	hypothetical protein	BCM95067.1	0	84.21%	
SURPRISE13_033	28793	29554	-	hypothetical protein	hypothetical protein	YP_009212879.1	6.00E-90	55.56%	
SURPRISE13_034	29674	30180	-	hypothetical protein	hypothetical protein	YP_009207838.1	4.00E-103	86.90%	
SURPRISE13_035	30251	30700	-	hypothetical protein	hypothetical protein	YP_009207839.1	2.00E-74	71.52%	
SURPRISE13_036	30888	31670	-	virion structure and maturation	head maturation protease	YP_009212882.1	2.00E-172	86.54%	PFAM:PF20034:Peptidase_S80
SURPRISE13_037	31691	32413	-	hypothetical protein	hypothetical protein	BCM95072.1	7.00E-168	95.00%	
SURPRISE13_038	32413	33846	-	hypothetical protein	hypothetical protein	YP_009207842.1	0	80.92%	
SURPRISE13_039	33860	34135	-	hypothetical protein	hypothetical protein	YP_009212885.1	4.00E-49	84.27%	
SURPRISE13_040	34219	38634	-	nucleic acid metabolism	RNA polymerase beta subunit	YP_009212886.1	0	91.91%	
SURPRISE13_041	38682	40298	-	nucleic acid metabolism	RNA polymerase beta subunit	BCM95076.1	0	95.91%	SUPFAM:SSF64484:beta_&_beta-prime_subunits_of_DNA_dependent_RNA-polymerase
SURPRISE13_042	40327	46515	+	virion structure and maturation	tail fiber protein	YP_009212888.1	0	77.61%	
SURPRISE13_043	46589	48484	+	hypothetical protein	hypothetical protein	BCM95078.1	0	91.13%	
SURPRISE13_044	48533	49711	-	hypothetical protein	hypothetical protein	BDD79326.1	0	81.38%	
SURPRISE13_045	49799	50326	-	hypothetical protein	hypothetical protein	YP_009207849.2	1.00E-117	91.18%	
SURPRISE13_046	50319	50894	-	hypothetical protein	hypothetical protein	YP_009212892.1	9.00E-105	86.02%	
SURPRISE13_047	50949	51464	-	hypothetical protein	hypothetical protein	YP_009207851.2	2.00E-93	77.11%	
SURPRISE13_048	51544	51981	+	hypothetical protein	hypothetical protein	BCM95083.1	9.00E-90	86.90%	
SURPRISE13_049	51978	52772	-	hypothetical protein	hypothetical protein	YP_009207853.1	7.00E-143	73.95%	
SURPRISE13_050	52769	53257	-	hypothetical protein	hypothetical protein	YP_009207854.1	1.00E-79	67.28%	
SURPRISE13_051	53322	54005	-	hypothetical protein	hypothetical protein	YP_009212897.1	2.00E-162	94.27%	
SURPRISE13_052	53995	54483	-	hypothetical protein	hypothetical protein	YP_009207856.1	1.00E-102	88.54%	
SURPRISE13_053	54577	55983	-	hypothetical protein	hypothetical protein	BAQ02578.2	0	92.95%	
SURPRISE13_054	56093	56971	+	hypothetical protein	hypothetical protein	YP_009207858.1	0	89.35%	
SURPRISE13_055	57040	57498	-	lysis	transglycosylase	YP_009207859.2	3.00E-72	72.73%	CDD:cd13400:LT_lagB-like
SURPRISE13_056	57615	58040	-	hypothetical protein	hypothetical protein	BDD79338.1	3.00E-57	74.34%	
SURPRISE13_057	58078	59667	-	nucleic acid metabolism	RNase-H family protein	BCM95092.1	0	89.79%	PFAM:PF00075:RNase_H
SURPRISE13_058	59719	60036	-	hypothetical protein	hypothetical protein	BDD79340.1	2.00E-43	62.16%	
SURPRISE13_059	60095	60592	-	hypothetical protein	hypothetical protein	BCM95094.1	1.00E-102	85.45%	
SURPRISE13_060	60702	62003	+	virion structure and maturation	virion structural protein	YP_009212906.1	0	89.38%	
SURPRISE13_061	62003	62638	+	hypothetical protein	hypothetical protein	BDD79343.1	4.00E-104	79.15%	
SURPRISE13_062	62702	63394	+	accessory/unknown function	concanavalin A-like lectin/glucanases superfamily protein	YP_009301713.1	7.00E-08	26.88%	SUPFAM:SSF49899:Concanavalin_A-like_lectins/glucanases
SURPRISE13_063	63391	64056	+	accessory/unknown function	concanavalin A-like lectin/glucanases superfamily protein	BDD79344.1	2.00E-83	61.76%%	SUPFAM:SSF49899:Concanavalin_A-like_lectins/glucanases
SURPRISE13_064	64059	64736	+	accessory/unknown function	concanavalin A-like lectin/glucanases superfamily protein	BDD79826.1	4.00E-06	30.77%%	SUPFAM:SSF49899:Concanavalin_A-like_lectins/glucanases
SURPRISE13_065	64783	67299	-	hypothetical protein	hypothetical protein	YP_009212910.1	0	71.24%	
SURPRISE13_066	67369	68370	+	virion structure and maturation	baseplate assembly protein	YP_009207868.1	0	81.98%	
SURPRISE13_067	68415	69692	-	hypothetical protein	hypothetical protein	YP_009212912.1	0	87.23%	
SURPRISE13_068	69778	71850	+	hypothetical protein	hypothetical protein	YP_009212913.1	0	75.65%	
SURPRISE13_069	71862	72983	+	virion structure and maturation	virion structural protein	YP_009212914.1	0	85.79%	
SURPRISE13_070	73057	73593	+	accessory/unknown function	cupin_2 family protein	YP_009207873.1	4.00E-94	73.03%	PFAM:PF078833:Cupin_2
SURPRISE13_071	73595	74659	+	accessory/unknown function	radical SAM domain-containing protein	YP_009212916.1	0	87.57%	CDD:cd01335:Radical_SAM
SURPRISE13_072	74666	75226	+	hypothetical protein	hypothetical protein	YP_009212917.1	4.00E-122	89.25%	
SURPRISE13_073	75237	76307	+	hypothetical protein	hypothetical protein	YP_009212918.1	0	77.53%	
SURPRISE13_074	76401	77852	+	hypothetical protein	hypothetical protein	BAQ02598.2	9.00E-172	62.50%	
SURPRISE13_075	77830	78930	+	hypothetical protein	hypothetical protein	YP_009212920.1	0	86.83%	
SURPRISE13_076	78981	80003	+	accessory/unknown function	radical SAM domain-containing protein	BDD79358.1	0	86.69%	CDD:cd01335:Radical_SAM
SURPRISE13_077	80000	80524	+	nucleic acid metabolism	dihydrofolate reductase	BCM95113.1	3.00E-92	70.53%	CDD:cd00209:DHFR
SURPRISE13_078	80527	81177	+	nucleic acid metabolism	dihydrofolate reductase	BCM95114.1	6.00E-48	40.18%	CDD:cd00209:DHFR
SURPRISE13_079	81131	82210	+	accessory/unknown function	radical SAM domain-containing protein	YP_009212924.1	0	92.16%	CDD:cd01335:Radical_SAM
SURPRISE13_080	82210	82755	+	accessory/unknown function	2OG-Fe(II) oxygenase family protein	YP_009207884.1	2.00E-126	92.82%	PFAM:PF13759:2OG-FeII_Oxy_5
SURPRISE13_081	82763	83707	+	accessory/unknown function	radical SAM domain-containing protein	BCM95117.1	0	90.45%	CDD:cd01335:Radical_SAM
SURPRISE13_082	83704	84273	+	accessory/unknown function	2OG-Fe(II) oxygenase family protein	YP_009207886.2	2.00E-82	62.98%	PFAM:PF13759:2OG-FeII_Oxy_5
SURPRISE13_083	84270	84938	+	hypothetical protein	hypothetical protein	YP_009212928.1	6.00E-135	81.45%	
SURPRISE13_084	84935	85516	+	hypothetical protein	hypothetical protein	YP_009207888.1	3.00E-114	77.72%	
SURPRISE13_085	85516	85935	+	hypothetical protein	hypothetical protein	BCM95121.1	6.00E-85	82.73%	
SURPRISE13_086	85970	86278	+	hypothetical protein	hypothetical protein	YP_009207891.1	3.00E-43	75.49%	
SURPRISE13_087	86333	87025	-	hypothetical protein	hypothetical protein	BCM95123.1	6.00E-131	77.19%	
SURPRISE13_088	87028	88290	-	hypothetical protein	hypothetical protein	BCM95123.1	7.00E-33	38.46%	
SURPRISE13_089	88413	89384	-	hypothetical protein	hypothetical protein	No Hits	-	-	
SURPRISE13_090	89447	89959	+	hypothetical protein	hypothetical protein	BDD79371.1	1.00E-39	48.17%	
SURPRISE13_091	89969	90439	+	hypothetical protein	hypothetical protein	BDD79371.1	5.00E-74	71.43%	
SURPRISE13_092	90436	91239	+	lysis	endolysin	BCM95126.1	4.00E-164	83.59%	PFAM:PF01471:PG_binding_1; PFAM:PF11860:Muramidase
SURPRISE13_093	91298	91813	+	hypothetical protein	hypothetical protein	WBF04692.1	1.00E-08	29.34%	
SURPRISE13_094	91866	92144	-	hypothetical protein	hypothetical protein	BCM95127.1	3.00E-55	88.04%	
SURPRISE13_095	92144	93727	-	hypothetical protein	hypothetical protein	BAQ02613.2	0	73.09%	
SURPRISE13_096	93743	94063	-	hypothetical protein	hypothetical protein	YP_009207899.1	3.00E-47	75.47%	
SURPRISE13_097	94044	94385	-	hypothetical protein	hypothetical protein	BDD79376.1	3.00E-58	78.76%	
SURPRISE13_098	94382	94879	-	hypothetical protein	hypothetical protein	YP_009212937.1	7.00E-82	75.76%	
SURPRISE13_099	94869	95450	-	hypothetical protein	hypothetical protein	YP_009212938.1	7.00E-125	87.05%	
SURPRISE13_100	95454	96308	-	hypothetical protein	hypothetical protein	YP_009207903.1	0	88.38%	
SURPRISE13_101	96332	98515	-	hypothetical protein	hypothetical protein	BAQ02619.2	0	87.76%	
SURPRISE13_102	98527	101529	+	virion structure and maturation	virion structural protein	YP_009207905.1	0	87.74%	
SURPRISE13_103	101618	102460	+	hypothetical protein	hypothetical protein	BCM95136.1	9.00E-50	71.90%	
SURPRISE13_104	102500	103177	-	hypothetical protein	hypothetical protein	WBF04845.1	4.00E-22	35.92%	
SURPRISE13_105	103202	103600	-	hypothetical protein	hypothetical protein	YP_009207908.2	6.00E-72	82.79%	
SURPRISE13_106	103662	105719	-	nucleic acid metabolism	NAD-dependent DNA ligase	YP_009207909.1	0	68.91%	PFAM:PF01653:DNA_ligase_aden
SURPRISE13_107	105878	106444	-	hypothetical protein	hypothetical protein	YP_009207911.1	2.00E-69	59.69%	
SURPRISE13_108	106431	106697	-	hypothetical protein	hypothetical protein	BCM95144.1	1.00E-51	90.48%	
SURPRISE13_109	106706	107434	-	hypothetical protein	hypothetical protein	YP_009212948.1	1.00E-139	76.86%	
SURPRISE13_110	107443	107709	-	hypothetical protein	hypothetical protein	BCM95146.1	2.00E-44	80.68%	
SURPRISE13_111	107778	108308	-	hypothetical protein	hypothetical protein	BCM95147.1	7.00E-94	71.59%	
SURPRISE13_112	108338	108544	-	hypothetical protein	hypothetical protein	BDD79394.1	4.00E-27	74.24%	
SURPRISE13_113	108629	109864	-	hypothetical protein	hypothetical protein	YP_009212953.1	0	65.62%	
SURPRISE13_114	109923	111377	-	accessory/unknown function	HAD-like domain-containing protein	YP_009207918.1	0	73.92%	CDD:cd01427:HAD-like
SURPRISE13_115	111381	112034	-	hypothetical protein	hypothetical protein	YP_009207919.1	4.00E-119	72.35%	
SURPRISE13_116	112039	112440	-	hypothetical protein	hypothetical protein	YP_009207920.2	4.00E-55	69.23%	
SURPRISE13_117	112578	113711	+	hypothetical protein	hypothetical protein	YP_009664398.1	1.00E-36	34.70%	
SURPRISE13_118	113711	114814	+	hypothetical protein	hypothetical protein	WCS68124.1	8.00E-33	34.31%	
SURPRISE13_119	114862	115995	-	nucleic acid metabolism	ribonucleotide reductase small subunit	YP_009207921.1	0	95.49%	PFAM:PF00268:Ribonuc_red_sm
SURPRISE13_120	116062	119082	-	nucleic acid metabolism	ribonucleotide reductase large subunit	YP_009212960.1	0	87.25%	PFAM:PF00317:Ribonuc_red_lgN; PFAM:PF02867:Ribonuc_red_lgC
SURPRISE13_121	119100	119345	-	nucleic acid metabolism	NrdH-redoxin family protein	YP_009212961.1	4.00E-51	92.59%	PFAM:PF00462:Glutaredoxin; CDD:cd02976:NrdH
SURPRISE13_122	119569	120156	-	hypothetical protein	hypothetical protein	BCM95157.1	4.00E-105	72.82%	
SURPRISE13_123	120231	122342	-	hypothetical protein	hypothetical protein	BCM95158.1	0	74.82%	
SURPRISE13_124	122350	124029	-	hypothetical protein	hypothetical protein	YP_009212964.1	0	92.13%	
SURPRISE13_125	124049	125197	-	hypothetical protein	hypothetical protein	YP_009212965.1	0	80.63%	
SURPRISE13_126	125458	127719	-	virion structure and maturation	major head protein	BDD79407.1	0	90.53%	
SURPRISE13_127	127832	128473	-	hypothetical protein	hypothetical protein	YP_009212967.1	9.00E-134	84.98%	
SURPRISE13_128	128703	130094	+	nucleic acid metabolism	DnaB-like replicative helicase	YP_009207930.2	0	90.71%	PFAM:divDNAB:PF20307
SURPRISE13_129	130149	131138	-	hypothetical protein	hypothetical protein	No Hits	-	-	
SURPRISE13_130	131217	131408	-	hypothetical protein	hypothetical protein	BDD79412.1	8.00E-26	80.70%	
SURPRISE13_131	131395	131796	-	hypothetical protein	hypothetical protein	YP_009212970.1	3.00E-72	78.20%	
SURPRISE13_132	131762	132124	-	hypothetical protein	hypothetical protein	BCM95168.1	4.00E-47	64.17%	
SURPRISE13_133	132133	132480	-	hypothetical protein	hypothetical protein	YP_009207934.1	6.00E-20	39.64%	
SURPRISE13_134	132484	133041	-	hypothetical protein	hypothetical protein	BDD79416.1	7.00E-33	36.02%	

SURPRISE13_135	133160	133480	-	hypothetical protein	hypothetical protein	BDD79417.1	7.00E-61	89.62%	
SURPRISE13_136	133507	133683	-	hypothetical protein	hypothetical protein	BCM95172.1	3.00E-24	75.86%	
SURPRISE13_137	133770	133931	-	hypothetical protein	hypothetical protein	YP_009207939.1	2.00E-18	71.15%	
SURPRISE13_138	134362	135402	-	hypothetical protein	hypothetical protein	YP_009207943.1	2.00E-142	63.26%	
SURPRISE13_139	135481	136770	-	hypothetical protein	hypothetical protein	BCM95175.1	2.00E-150	55.80%	
SURPRISE13_140	136773	137120	-	hypothetical protein	hypothetical protein	YP_009207945.1	1.00E-55	74.78%	
SURPRISE13_141	137120	137347	-	hypothetical protein	hypothetical protein	YP_009207946.2	2.00E-32	73.33%	
SURPRISE13_142	137494	138504	-	hypothetical protein	hypothetical protein	YP_009212981.1	5.00E-148	59.52%	
SURPRISE13_143	138452	138961	-	hypothetical protein	hypothetical protein	BAU71407.1	1.00E-75	64.12%	
SURPRISE13_144	139030	140442	-	hypothetical protein	hypothetical protein	BDD79426.1	0	72.77%	
SURPRISE13_145	140554	141138	-	hypothetical protein	hypothetical protein	BDD79427.1	4.00E-99	71.13%	
SURPRISE13_146	141211	141633	-	hypothetical protein	hypothetical protein	YP_009207951.1	1.00E-72	77.30%	
SURPRISE13_147	141645	142106	-	hypothetical protein	hypothetical protein	YP_009207952.1	1.00E-25	39.42%	
SURPRISE13_148	142197	142565	-	hypothetical protein	hypothetical protein	BDD79430.1	6.00E-59	71.43%	
SURPRISE13_149	142609	142965	-	hypothetical protein	hypothetical protein	YP_009207953.1	3.00E-74	90.68%	
SURPRISE13_150	143045	143551	-	hypothetical protein	hypothetical protein	BDD79432.1	3.00E-76	72.97%	
SURPRISE13_151	143541	143789	-	hypothetical protein	hypothetical protein	YP_009207955.1	7.00E-41	82.93%	
SURPRISE13_152	143801	144088	-	hypothetical protein	hypothetical protein	WP_168306181.1	2.00E-15	44.00%	
SURPRISE13_153	144113	145456	-	hypothetical protein	hypothetical protein	YP_009212989.1	0	61.61%	
SURPRISE13_154	145464	145778	-	hypothetical protein	hypothetical protein	BDD79435.1	3.00E-14	46.15%	
SURPRISE13_155	145753	146613	-	accessory/unknown function	QueC-like queuosine biosynthesis family protein	YP_009207957.1	1.00E-128	68.20%	F06508:Queuosine_biosynthesis_protein_QueC; SUPFAM:SSF52402:Adenine_nucleotide_alpha_hydrolases-like
SURPRISE13_156	146615	147208	-	hypothetical protein	hypothetical protein	YP_009207958.1	4.00E-41	47.57%	
SURPRISE13_157	147220	147660	-	hypothetical protein	hypothetical protein	BCM95192.1	2.00E-69	69.66%	
SURPRISE13_158	147666	147860	-	hypothetical protein	hypothetical protein	YP_009212993.1	4.00E-29	78.12%	
SURPRISE13_159	147915	148553	-	hypothetical protein	hypothetical protein	BCM95195.1	4.00E-89	79.91%	
SURPRISE13_160	148580	149131	-	hypothetical protein	hypothetical protein	YP_009212995.1	5.00E-110	82.51%	
SURPRISE13_161	149345	149824	-	hypothetical protein	hypothetical protein	YP_009212996.1	2.00E-64	85.84%	
SURPRISE13_162	149796	150278	-	accessory/unknown function	HAD-like superfamily protein	BCM95198.1	7.00E-95	81.99%	SUPFAM:SSF56784:HAD-like
SURPRISE13_163	150371	150841	-	hypothetical protein	hypothetical protein	YP_009212999.1	1.00E-96	87.26%	
SURPRISE13_164	150852	151736	-	hypothetical protein	hypothetical protein	YP_009213000.1	2.00E-159	72.79%	
SURPRISE13_165	151737	152270	-	hypothetical protein	hypothetical protein	YP_009213001.1	5.00E-95	74.01%	
SURPRISE13_166	152368	152601	+	hypothetical protein	hypothetical protein	No Hits	-	-	
SURPRISE13_167	152873	154159	-	nucleic acid metabolism	thymidylate synthase	YP_009207968.1	0	73.36%	CDD:cd00351:TS_Pyrimidine_Hmase
SURPRISE13_168	154222	154602	-	hypothetical protein	hypothetical protein	YP_009207969.1	9.00E-62	75.40%	
SURPRISE13_169	155196	155483	-	hypothetical protein	hypothetical protein	No Hits	-	-	
SURPRISE13_170	155493	156779	-	hypothetical protein	hypothetical protein	YP_009207970.1	0	73.93%	
SURPRISE13_171	157029	157442	-	accessory/unknown function	DUF1353 family protein	BDD79455.1	6.00E-80	82.48%	PFAM:PF07087:DUF1353
SURPRISE13_172	157524	158195	-	hypothetical protein	hypothetical protein	YP_009207972.1	3.00E-134	82.81%	
SURPRISE13_173	158211	162191	-	virion structure and maturation	tail fiber protein	BDD79457.1	0	68.61%	
SURPRISE13_174	162244	163302	-	hypothetical protein	hypothetical protein	BCM95213.1	0	89.49%	
SURPRISE13_175	163408	163710	-	hypothetical protein	hypothetical protein	BCM95214.1	3.00E-41	71.43%	
SURPRISE13_176	163768	164163	-	hypothetical protein	hypothetical protein	WOL24507.1	9.00E-25	47.46%	
SURPRISE13_177	164228	164695	-	hypothetical protein	hypothetical protein	BDD79461.1	5.00E-46	50.31%	
SURPRISE13_178	164824	166206	-	hypothetical protein	hypothetical protein	YP_009207978.1	0	83.48%	
SURPRISE13_179	166203	166907	-	hypothetical protein	hypothetical protein	YP_009213013.1	5.00E-147	84.19%	
SURPRISE13_180	166904	168253	-	virion structure and maturation	virion structural protein	YP_009213014.1	0	85.08%	
SURPRISE13_181	168262	169818	-	hypothetical protein	hypothetical protein	BDD79465.1	0	84.84%	
SURPRISE13_182	169853	170269	-	accessory/unknown function	DUF6527 family protein	YP_009213016.1	2.00E-85	82.61%	PFAM:PF20137:DUF6527
SURPRISE13_183	170350	170610	-	hypothetical protein	hypothetical protein	YP_009213017.1	6.00E-23	59.34%	
SURPRISE13_184	170613	171923	-	accessory/unknown function	phiKZ-like phage internal head family protein	YP_009207983.1	0	78.44%	PFAM:PF12699:phiKZ_IP
SURPRISE13_185	172031	173344	-	accessory/unknown function	phiKZ-like phage internal head family protein	YP_009213019.1	0	83.11%	PFAM:PF12699:phiKZ_IP
SURPRISE13_186	173479	174060	-	virion structure and maturation	virion structural protein	YP_009207985.1	2.00E-122	86.08%	
SURPRISE13_187	174060	174950	-	hypothetical protein	hypothetical protein	YP_009207986.1	0	89.86%	
SURPRISE13_188	174968	176065	-	hypothetical protein	hypothetical protein	YP_009213022.1	0	91.80%	
SURPRISE13_189	176098	177366	+	hypothetical protein	hypothetical protein	YP_009213023.1	0	92.42%	
SURPRISE13_190	177381	180299	+	hypothetical protein	hypothetical protein	YP_009207989.1	0	86.51%	
SURPRISE13_191	180353	182170	-	hypothetical protein	hypothetical protein	YP_009213025.1	0	71.03%	
SURPRISE13_192	182182	182658	-	hypothetical protein	hypothetical protein	YP_009207991.1	3.00E-94	84.18%	
SURPRISE13_193	182675	183985	-	hypothetical protein	hypothetical protein	BAQ02706.2	0	86.93%	
SURPRISE13_194	184191	186170	+	nucleic acid metabolism	DNA polymerase	YP_009207993.2	0	90.29%	SUPFAM:SSF53098:Ribonuclease_H-like
SURPRISE13_195	186937	187509	-	accessory/unknown function	concanavalin A-like lectins/glucanases superfamily protein	BAQ02709.2	8.00E-41	42.56%	SUPFAM:SSF49900:concanavalin_A-like_lectins/glucanases
SURPRISE13_196	187579	188274	-	nucleic acid metabolism	thymidylate kinase	YP_009207996.2	5.00E-138	79.48%	PFAM:PF02223:Thymidylate_kin
SURPRISE13_197	188384	189154	-	hypothetical protein	hypothetical protein	BDD79482.1	7.00E-166	87.50%	
SURPRISE13_198	189197	189922	-	hypothetical protein	hypothetical protein	YP_009207998.1	2.00E-140	85.00%	
SURPRISE13_199	190120	190503	-	hypothetical protein	hypothetical protein	YP_009207999.2	1.00E-71	79.53%	
SURPRISE13_200	190566	192089	-	hypothetical protein	hypothetical protein	YP_009208000.1	0	85.04%	
SURPRISE13_201	192102	192812	-	hypothetical protein	hypothetical protein	YP_009208001.1	2.00E-99	71.57%	
SURPRISE13_202	192926	193822	+	hypothetical protein	hypothetical protein	BAQ02719.2	0	87.25%	
SURPRISE13_203	193815	195113	+	hypothetical protein	hypothetical protein	BCM95243.1	0	92.13%	
SURPRISE13_204	195140	196129	+	hypothetical protein	hypothetical protein	YP_009208004.2	9.00E-101	66.52%	
SURPRISE13_205	196194	197036	-	hypothetical protein	hypothetical protein	YP_009208005.1	1.00E-134	67.38%	
SURPRISE13_206	197060	197464	-	hypothetical protein	hypothetical protein	YP_009213044.1	4.00E-88	92.54%	
SURPRISE13_207	197466	197741	-	accessory/unknown function	acyl carrier protein-like superfamily protein	BCM95247.1	1.00E-40	76.92%	SUPFAM:SSF47336:ACP-like
SURPRISE13_208	198528	199283	-	hypothetical protein	hypothetical protein	BCM95248.1	5.00E-27	45.22%	
SURPRISE13_209	199496	199834	-	hypothetical protein	hypothetical protein	DAV81402.1	2.00E-42	59.43%	
SURPRISE13_210	199836	200213	-	accessory/unknown function	DUF3307 family protein	YP_009208009.1	1.00E-54	65.60%	PFAM:PF11750:DUF3307
SURPRISE13_211	200515	201024	-	hypothetical protein	hypothetical protein	BCM95251.1	2.00E-95	78.11%	
SURPRISE13_212	201027	201716	-	hypothetical protein	hypothetical protein	YP_009208013.2	9.00E-132	76.75%	
SURPRISE13_213	201732	202127	-	hypothetical protein	hypothetical protein	WP_054432600.1	3.00E-51	62.60%	
SURPRISE13_214	202251	203285	-	hypothetical protein	hypothetical protein	BAQ02732.2	4.00E-106	48.82%	
SURPRISE13_215	203357	203590	-	hypothetical protein	hypothetical protein	No Hits	-	-	
SURPRISE13_216	203737	204813	-	hypothetical protein	hypothetical protein	No Hits	-	-	
SURPRISE13_217	204952	206562	-	nucleic acid metabolism	DNA helicase	BCM95257.1	0	85.07%	CDD:cd18785:SF2_C
SURPRISE13_218	206669	208675	-	hypothetical protein	hypothetical protein	BCM95259.1	0	88.62%	
SURPRISE13_219	208681	210891	-	nucleic acid metabolism	RNA polymerase beta subunit	YP_009213058.1	0	91.80%	PFAM:PF00562:RNA_pol_Rpb2_6
SURPRISE13_220	211016	211651	-	accessory/unknown function	GIY-YIG endonuclease superfamily protein	UQT03168.1	2.00E-13	29.35%	SUPFAM:SSF8277:GIY-YIG_endonuclease
SURPRISE13_221	211721	212062	+	hypothetical protein	hypothetical protein	BCM95261.1	9.00E-71	87.61%	
SURPRISE13_222	212122	213744	-	hypothetical protein	hypothetical protein	YP_009213060.1	0	83.89%	
SURPRISE13_223	213758	215209	-	hypothetical protein	hypothetical protein	YP_009208022.1	0	83.64%	
SURPRISE13_224	215227	216792	-	hypothetical protein	hypothetical protein	YP_009208023.2	0	90.40%	
SURPRISE13_225	216920	217687	-	hypothetical protein	hypothetical protein	YP_009208024.1	5.00E-110	78.01%	
SURPRISE13_226	217753	218589	-	hypothetical protein	hypothetical protein	YP_009213064.1	0	92.45%	
SURPRISE13_227	218586	218777	-	hypothetical protein	hypothetical protein	No Hits	-	-	
SURPRISE13_228	218774	219259	-	hypothetical protein	hypothetical protein	YP_009213065.1	5.00E-50	52.53%	
SURPRISE13_229	219266	220417	-	nucleic acid metabolism	calcineurin-like phosphoesterase family protein	YP_009208027.2	0	88.02%	PFAM:PF00149:Calcineurin-like_phosphoesterase
SURPRISE13_230	220371	221090	-	hypothetical protein	hypothetical protein	YP_009213067.1	8.00E-145	82.35%	
SURPRISE13_231	221087	221500	-	hypothetical protein	hypothetical protein	BCM95271.1	4.00E-74	80.43%	
SURPRISE13_232	221562	222224	-	hypothetical protein	hypothetical protein	YP_009208030.1	5.00E-140	89.09%	
SURPRISE13_233	222224	223855	-	nucleic acid metabolism	RNA polymerase beta subunit	YP_009208031.2	0	94.99%	
SURPRISE13_234	224016	224210	-	hypothetical protein	hypothetical protein	YP_009213071.1	2.00E-05	33.33%	
SURPRISE13_235	224305	226248	-	phage nuclear shell	hypothetical protein	YP_009208033.2	0	92.13%	
SURPRISE13_236	226548	227627	+	hypothetical protein	hypothetical protein	YP_009213073.1	0	80.78%	
