# Supplementary material for: A flagella-dependent Burkholderia jumbo phage controls rice seedling rot and steers Burkholderia glumae toward reduced virulence in rice seedlings
Source: mBio. 2025 Jan 27;16(3):e02814-24. doi: 10.1128/mbio.02814-24 (PMC11898562; doi:10.1128/mbio.02814-24)
Supplement: Figure S1 — Swimming motility of additional fully and partially S13-resistant B. glumae LMG 2196 isolates. [file mbio.02814-24-s0001.pdf]

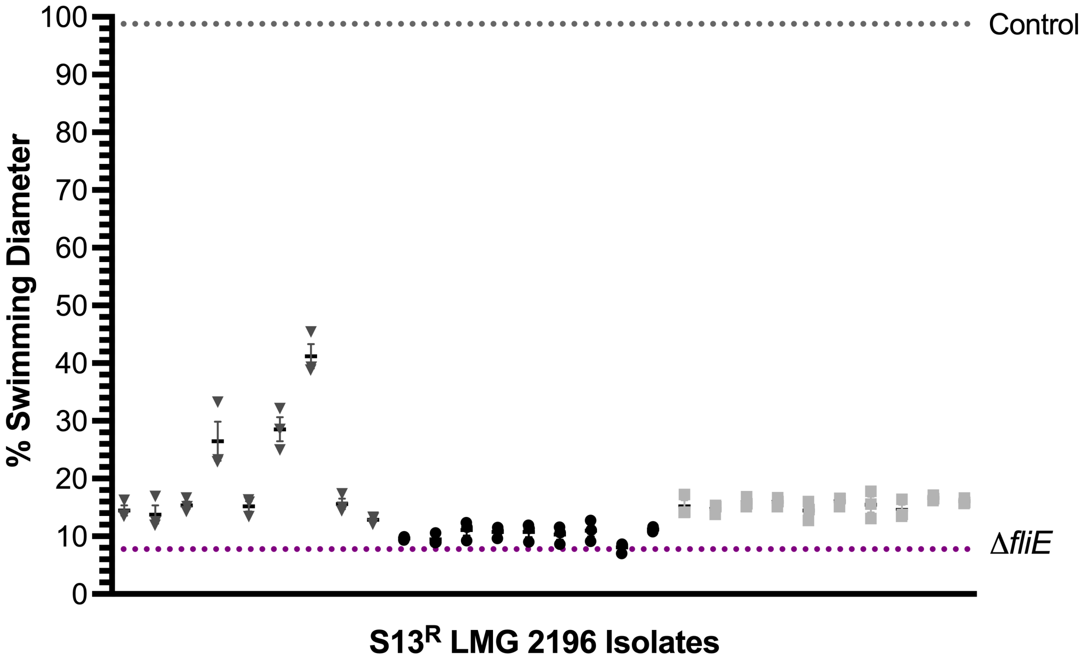

Figure S1: Swimming motility of additional fully and partially S13-resistant (S13^R) *B. glumae* LMG 2196 isolates**.** The swim zones of 29 S13^R isolates were measured after 24 hours at 30°C and normalized to WT LMG 2196. The purple line represents the average % swimming diameter of LMG 2196 Δ*fliE* , while the grey line represents the average % swimming diameter of evolved control isolates of LMG 2196. Colonies derived from the same S13^R mutant isolation experiment are grouped by symbols, with a total of three isolations represented. Error bars represent standard error of the mean (SEM) of three replicates per isolate.
